# TRPM7 silencing modulates glucose metabolic reprogramming to inhibit the growth of ovarian cancer by enhancing AMPK activation to promote HIF-1α degradation

**DOI:** 10.1186/s13046-022-02252-1

**Published:** 2022-01-31

**Authors:** Yongchang Chen, Lu Liu, Longzheng Xia, Nayiyuan Wu, Ying Wang, He Li, Xue Chen, Xiaoye Zhang, Zhaoyi Liu, Miaochen Zhu, Qianjin Liao, Jing Wang

**Affiliations:** 1 Hunan clinicaI research center in gynecologic cancer, Hunan Key Laboratory of Cancer Metabolism, The Affiliated Cancer Hospital of Xiangya School of Medicine, Central South University and Hunan Cancer Hospital, The Affiliated Cancer Hospital of Xiangya School of Medicine, Central South University, Changsha, 410013 Hunan China; 2 University of South China, Hengyang, 421001 Hunan China

**Keywords:** TRPM7, AMPK, HIF-1α, Glycolysis, Oxidative phosphorylation, Ubiquitination, Ovarian cancer

## Abstract

**Background:**

Tumor cell metabolic reprogramming is crucial for the malignant behavior of cancer cells by promoting their proliferation. However, little is known on how transient receptor potential 7 (TRPM7) modulates metabolic reprogramming in ovarian cancer.

**Methods:**

The effects of TRPM7 silencing on transcriptome profile, glucose uptake, lactic acid production, extracellular acidification rate (ECAR), oxygen consumption rate (OCR), intracellular ROS and ATP levels, and NAD+/NADH ratios in ovarian cancer cells were examined. The impacts of TRPM7 silencing on the levels of glycolysis-related HK2, PDK1 and oxidative phosphorylation (OXPHOS)-related IDH3B and UQCRC1, HIF-1α expression and AMPK phosphorylation were determined in ovarian cancer. The effect of AMPK activity on HIF-1α ubiquitination degradation was investigated in ovarian cancer cells.

**Results:**

Compared with the control, TRPM7 silencing suppressed the proliferation of ovarian cancer cells by shifting preferable glycolysis to OXPHOS. In parallel, TRPM7 silencing decreased the glucose uptake of tumor-bearing mice and TRPM7 levels were negatively correlated with IDH3B and UQCRC1, but positively with HK2 and PDK1 expression in ovarian cancer tissues. Mechanistically, TRPM7 silencing significantly increased AMPK phosphorylation and decreased HIF-1α protein levels in ovarian cancer, particularly in HIF-1α silencing cells. The shifting from glycolysis to OXPHOS by TRPM7 silencing was abrogated by HIF-1α over-expression and impaired by inhibiting AMPK activity in ovarian cancer cells. Moreover, enhanced AMPK activation inhibited glycolysis, which was abrogated by HIF-1α over-expression in ovarian cancer cells. Moreover, the enhanced AMPK activation promoted HIF-1α ubiquitination degradation.

**Conclusions:**

TRPM7 silencing enhanced AMPK activation to shift glycolysis to oxidative phosphorylation by promoting HIF-1α ubiquitination degradation in ovarian cancer. Hence, TRPM7 may be a therapeutic target for intervention of ovarian cancer.

**Supplementary Information:**

The online version contains supplementary material available at 10.1186/s13046-022-02252-1.

## Background

Ovarian cancer is a common gynecological malignancy in women. Its prevalence and incidence remain high, affecting many women in the world. Currently, the standard therapies for ovarian cancer include surgical resection and chemotherapy, but ovarian cancer patients still have a 5-year survival rate of < 50% due to the tumor recurrence and distant metastasis [1–3]. Hence, discovering the molecular pathogenesis, and novel prognostic biomarkers and therapeutic targets is of high significance in prognosis and treatment of ovarian cancer.

It is well known that the majority of healthy cells prefer glucose metabolism by oxidative phosphorylation (OXPHOS) to produce high levels of ATP and reactive oxygen species (ROS), which can cause cell injury. In contrast, malignant tumor cells can reprogram their energy metabolism and escape from immune destruction [4, 5]. Tumor cells can alter their glucose metabolism by preferring glycolysis even in a normoxic environment to support their malignant behaviors, such as proliferation, invasion and metastasis, called the “Warburg effect” [6–8]. However, it is still unclear how and what factors regulate the Warburg effect and OXPHOS to support the growth of ovarian cancer.

The hypoxia inducible factor (HIF)-1α and adenosine 5′-monophosphate (AMP)-activated protein kinase (AMPK) signal pathways are the major regulators of glycolysis and OXPHOS, and they are crucial for the metabolic reprogramming in tumor cells [9, 10]. AMPK is an evolutionarily conserved serine / threonine kinase. AMPK can be activated by many factors, such as AMP / ATP and ADP / ATP ratios [11, 12] and intracellular calcium through CaMKK2-mediated phosphorylation [13–15]. HIF-1α is commonly expressed by mammalian cells, but it is rapidly degraded by the intracellular oxygen-dependent ubiquitin protease degradation pathway in a normoxygenic condition. HIF-1α expression is significantly up-regulated and HIF-1α protein stabilizes in a hypoxic condition. The AMPK and HIF pathways can also modulate the expression and function of several transcription factors, such as FoxO, NF-κB, NRF2, and p53, as well as protein kinases and other factors, including mTOR, ULK1, HDAC5, and SIRT1 [16]. However, little is known on how the AMPK and HIF pathways modulate the metabolic reprogramming in ovarian cancer.

Transient receptor potential melastatin 7 (TRPM7) is a unique cation channel protein, and functionally responsible for transportation of cations (such as calcium, magnesium, and others), thereby increasing [Ca^2+^]i levels. In addition, TRPM7 has a kinase activity and can activate itself and other substrates, dependent on ATP. TRPM7 expression is up-regulated in a variety of tumors and can enhance malignant behaviors of cancer cells [17]. Our previous study has revealed that up-regulated TRPM7 expression is related to pelvic lymph node metastasis and poor prognosis of human ovarian cancer and inhibition of TRPM7 can suppress the invasion and metastasis of ovarian cancer cells through attenuating the Ca^2+^-PI3K/AKT signaling [18]. Interestingly, inhibition of TRPM7 can promote HIF-1α degradation in prostate cancer cells [19]. Given that glycolysis inhibition is associated with HIF-1α degradation we hypothesize that TRPM7 activation may modulate the HIF-1α/AMPK signaling to regulate glucose metabolism and promote ovarian cancer cell proliferation.

This study investigated the impacts of TRPM7 silencing on ovarian cancer cell proliferation, glucose metabolism and the AMPK / HIF-1α signaling. We found that TRPM7 silencing enhanced OXPHOX, inhibited glucose uptake, glycolysis, lactic acid production and suppressed the proliferation of ovarian cancer cells by enhancing AMPK activation and HIF-1α degradation. Together, our findings indicate that TRPM7 is a novel therapeutic target and may shed new light on the regulation of metabolic reprogramming and pathogenesis of ovarian cancer.

## Materials and methods

### Specific reagents

The specific reagents induced antibodies against Hexokinase II (ab209847), PDK1 (ab110025), IDH3B (ab247089), TRPM7 (ab109438), UQCRC1 (ab223746), H3 (ab1791), rabbit anti-PKM2 (ab85555), goat anti-rabbit IgG (ab6721) and goat anti-mouse IgG (ab6728, Abcam, Cambridge, USA), phospho-AMPKα (#2535, Cell Signaling Technology, Saint Louis, USA), Alexa Fluor488-conjugated goat anti-rabbit IgG (#35552), Alexa Fluor594-conjugated goat anti-mouse IgG (#35560, Invitrogen, Waltham, USA), compounds of Dorsomorphin (Compound C) 2HCl (S7306) and metformin HCl (S1950, Selleckchem, Houston, USA) and DAPI staining solution (C1005, Beyotime, Beijing, China).

### Patients

We recruited 60 ovarian cancer patients without prior radiotherapy and chemotherapy in the Department of Gynecology, Cancer Hospital, Xiangya Medical College of Central South University of China. We collected surgical ovarian specimens from the patients when they underwent a surgery for removal of the tumor. Individual patients singed the written informed consent. The experiments were approved by the Joint Ethics Committee of Hunan Cancer Hospital and Affiliated Tumor Hospital of Xiangya Medical College of Central South University of China (approval number: KYJJ-2019-001).

### Cells and culture

Human ovarian cancer SKOV3 and HO8910 cells were provided by the Cancer Institute, Central South University and they were identified by STR. SKOV3 and HO8910 cells were cultured in RPMI1640 medium containing 10% fetal bowel serum (FBS, Life Technologies, Carlsbad, USA) at 37 °C in 5% CO_2_. Some cells were cultured in a hypoxic incubator of 1% O_2_, 5% CO_2_, and 94% N_2_ at 37 °C.

### Sequencing of mRNAs

Total RNA was extracted from SKOV3 sh-control and SKOV3 sh-TRPM7 cells and the samples (1–2 μg/each) were used for generation of libraries using the KAPA Stranded RNA-Seq Library Prep Kit (Illumina), according to the manufacturer’s protocol by KangChen BioTech, China. The barcoded libraries were purified and quantified using an Agilent 2100 bioanalyzer. The mixed libraries of different samples were denatured with 0.1 M NAOH, diluted to 8 pM and amplified in situ on TruSeq SR Cluster Kit v3-cBot-HS (#GD-401-3001, Illumina). The samples were sequenced at 2 × 150 bp in an Illumina X-ten/NovaSeq sequencer. The levels of gene expression were measured as fragments per kilobase per million reads (FPKM) and the DEGs were defined when a fold change ≥2.0 and a *P*-value of < 0.05.

### Bioinformatics

We analyzed the DEGs between the sh-control (C) and sh-TRPM7 (T) groups using Gene Set Enrichment Analysis (GSEA, GSEA v4.0.3 for windows) (http://software.broadinstitute.org/gsea/index.jsp) [20]. First, we analyzed the DEGs in the biological signaling using the MSigDB (Molecular Signatures Database) (http://software.broadinstitute.org/gsea/msigdb) with 1000-rounds. The identified DEGs were defined with a false discovery rate (FDR) < 0.25 and a family-wise error rate (FWR) < 0.05. Second, the DEGs were subjected to the Gene Ontology (GO) analysis organism (http://www.geneontology.org), particularly in Biological Process, Cellular Component and Molecular Function. The DEGs in the top GO lists were analyzed by Fisher’s exact test with a *P*-value of ≤0.05. Finally, the DEGs were analyzed for their pathways using the KEGG with a *P*-value of < 0.05.

### Immunohistochemistry (IHC)

The expression levels of specific proteins in ovarian cancer tissues were characterized by IHC using a kit (CW2069, CoWin Biosciences). Briefly, individual paraffin-embedded tissue sections (3 μm) were treated with 3% bowel serum albumin (BSA) and underwent routine-antigen retrieval. The sections were probed with mouse anti-Hexokinase II (1: 500), anti-PDK1 (1: 600), anti-IDH3B (1: 2000), anti-phospho-AMPKα (1: 100), anti-TRPM7 (1: 700), UQCRC1 (1: 400) or control mouse or rabbit IgG at 4 °C overnight. The sections were cultured with *horseradish peroxidase* (HRP)-conjugated detection antibodies (15000) and reacted with DAB. The sections were counter-stained with hematoxylin. The immune staining signals were photoimaged under a light microscope and semi-quantitatively evaluated by two pathologists in a blinded manner, according to the percentages of positively stained cells and the intensity of IHC signals. The percentages of positively stained cells were scored as 0: < 5%; 1: 6–25%; 2: 26 -50%; 3: > 50%. The intensity of IHC signals was quantified as 0: colorless; 1: light yellow; 2: brown yellow; 3: dark brown. A final score in each image was achieved as the intensity score × percentage score and the levels of protein expression were defined as 0: no expression; 1–4: low levels of its expression; 5–9: high levels of its expression.

### Transduction and transfection

The lentiviruses for TRPM7 knockdown, and HIF-1α over-expression or knockdown were purchased from Genepharma (shanghai, China). SKOV3 and HO8910 cells (5 × 10^5^/well) were cultured overnight and transduced with lentivirus at a MOI of 5 for expression of Con-sh, TRPM7-sh1, TRPM7-sh2, TRPM7-sh3, TRPM7-sh4, HIF-1α-sh or HIF-1α. The target sequences are shown in the Supplementary Table 1. After 48 h of incubation, their total RNAs and proteins were extracted for verification of gene silencing or over-expression. Some cells were cultured in the presence of G418 (500 μg/ml, Biofrox, Germany) for 14 days to generate stable gene silencing or over-expressing cells. Subsequently, we characterized the cell clones by quantitative real-time PCR (RT-qPCR) and Western blot.

### Immunofluorescence

SKOV3-Con-sh, SKOV3-TRPM7-sh, HO8910-Con-sh and HO8910-TRPM7-sh cells were stained with rabbit anti-Hexokinase II (1: 100), mouse anti-PDK1(1: 100), rabbit anti-IDH3B (1: 100), mouse anti-UQCRC1 (1: 70), rabbit anti-PKM2 (1: 50). Subsequently, they were reacted with Alexa Fluor488-goat anti-rabbit IgG and Alexa Fluor594-goat anti-mouse IgG, and nuclear-stained with DAPI. The cells were examined under a fluorescent microscope.

### RT-qPCR

We extracted total RNAs from each specimen or group of cells using Trizol reagent, and reversely transcribed them into cDNA using Revert Aid First Strand cDNA Synthesis Kit (K1622, Thermoscientific, USA). We quantified the relative levels of target gene to Tubulin mRNA transcripts by RT-qPCR using the FastStart Essential DNA Green Master kit (06924204001, Lifescience, Roche, Mannheim, Germany) and specific primers in the RocheLightCycler® 96 instrument and software (05815916001, Lifescience). The sequences of primers are shown in the Supplementary Table 2. We performed the PCR reactions in triplicate and analyzed the data using the 2^-ΔΔCt^ method.

### Western blot

We performed Western blot assays to quantify the relative levels of target protein expression in different groups of cells [21]. Briefly, we performed sodium dodecyl sulfate-polyacrylamide gel electrophoresis (SDS-PAGE) to separate the cell lysate proteins (30–50 μg/lane) using 10% gels and transferred them onto polyvinylidene difluoride (PVDF) membranes. We treated the membranes with 5% BSA in TBST and probed the membranes overnight at 4 °C with antibodies against HK2, PDK1, IDH3B, UQCRC1, AMPK, phosphor-AMPK, TRPM7 and α-Tubulin. Subsequently, we incubated the membranes with HRP-labeled second antibodies and developed the blots with the ECL substrate (32,109, ThermoScientific). Finally, we quantified the relative levels of individual proteins to α-Tubulin using the ImageJ software (Madison, USA).

### Animal experiments and PET/CT study

We obtained female BALB/c nude mice (6-week-old, ~ 20 g) from SLA Laboratory Animal (Changsha, China) and maintained them in a specific pathogen-free room. In the first set of experiments, we injected each mouse subcutaneously with five millions of SKOV3-Con-sh or SKOV3-TRPM7-sh cells in 0.15 mL of saline. Four weeks later, we dissected, weighed, photoimaged the tumors. In another set of experiments, we injected each mouse subcutaneously with 5 × 10^6^ SKOV3 cells. Three weeks later, the experimental group of mice were intraperitoneally injected 50 mg/kg carvacrol (an inhibitor of TRPM7, Sigma-Aldrich, 282,197) in DMSO vehicle daily for 7 consecutive days while the control mice received vehicle alone. At the end of treatment, we used PET/CT to measure the tumors in mice and euthanized them. We dissected, weighed and frozen tumors. Subsequently, we fixed some tumors from each group of mice in 10% formalin and paraffin-embedded them as well as froze some tumors in liquid nitrogen for subsequent experiments. An additional figure shows this in more detail [see Supplementary Fig. 1]. To perform the PET/CT imaging, we fasted the mice overnight and injected individual mice intravenously with approximately 200 ± 10 μCi 18-fluoro-6-deoxy-glucose (FDG, Wuhan Union Hospital PET Center, Wuhan, China). One hour later, we anesthetized the mice with 2% isoflurane and imaged them using PET with a static mode of 10 min and using CT scan of normal mode in the TransPET Discoverist 180 system (Raycan Technology, Suzhou, China). We reconstructed the PET images using the three-dimensional (3D) OSEM method with a voxel size of 0.5 × 0.5 × 0.5 mm3. We reconstructed the CT images using FDK algorithm with 256 × 256 × 256 matrix. We displayed the images with software Carimas (Turku PET Center, Turku, Finland). We calculated the mean standardized uptake value (SUV) using the following formula: mean pixel value with the decay-corrected region-of-interest activity (μCi /kg)/(injected dose [μCi]/weight [kg]). The experimental protocols were approved by the Animal Research and Care Committee of our hospital (approval number: 2019–014).

### Cell proliferation

The cell proliferation was tested by EdU assay, using the BeyoClick™ EdU-594 Cell Proliferation Assay Kit (Byotime), per the manufacturer’s protocol. Briefly, the different groups of cells (3 × 10^5^ cells/well) were cultured in 6-well plates for 48 h and labeled in triplicate with EdU and DAPI. The fluorescent signals were captured under a fluorescence microscope (Zeiss, Germany).

### Intracellular ROS levels

The contents of intracellular ROS in each group of cells were quantified using Reactive Oxygen Species Assay Kit (Byotime), per the manufacturer’s protocol. The frequency of fluorescence-positive cells was quantified by flow cytometry using CellQuest software.

### Measurement of glucose uptake and lactate production

The impact of TRPM7 silencing on glucose uptake and lactate production in each group of ovarian cancer cells was tested using the glucose uptake colorimetric assay kit and lactate colorimetric assay kit (Biovision, USA), following the manufacturer’s protocols.

### Seahorse assay

We performed seahorse assays to quantitatively measure ECAR and OCR of individual groups of cells using Seahorse XF Glycolysis Stress Test Kit and Seahorse XF Cell Mito Stress Test Kit in the Seahorse XFe 96 Extracellular Flux Analyzer (Seahorse Bioscience, USA), following the manufacturer’s protocols. Briefly, individual groups of cells (1 × 10^4^ cells/well) were cultured into a Seahorse XF 96-well microplate and their baseline measures were determined. Subsequently, the cells were treated sequentially with glucose, oligomycin (the oxidative phosphorylation inhibitor), and 2-DG (the glycolytic inhibitor) at indicated dose and time points for measurement of ECAR. Similarly, the cells were treated sequentially with oligomycin, oxidative phosphorylation FCCP (p-trifluoromethoxy carbonyl cyanide phenylhydrazone, the reversible inhibitor), and rotenone/antimycin A (Rote/AA, the mitochondrial complex I inhibitor and the mitochondrial complex III inhibitor, respectively). Data were analyzed by Seahorse XF-96 Wave software and expressed as pmols/min for OCR and mpH/min for ECAR, respectively.

### ATP assay

The levels of ATP production in each group of cells were determined by ATP assays using ATP Assay Kit (Byotime). Briefly, individual groups of cells were suspended in an ice-cold ATP-releasing buffer, and centrifuged. Individual supernatants or standard (100 μL each) were mixed with 100 μL ATP detection solution and analyzed using the Dual-Luciferase Reporter Assay System (Promega). After normalized with protein concentrations, the luminescent signals were used to quantify the levels of ATP, according to the standard curve prepared with known concentrations (1 nM - 1 mM) of ATP.

### In vivo ubiquitination assays

The impact of TRPM7 silencing on the stability of HIF-1α in ovarian cancer cells was examined by in vivo ubiquitination assays. Briefly, the different groups of cells were treated in triplicate with CC (20 μM) under a normoxic or hypoxic condition for 1 day. The cells were treated with MG132 (10 μM) for 8 h, and the levels of HIF-1α ubiquitination were determined by IP with anti-HIF-1α antibody, followed by Western blot using anti-Ub antibody (10201–1-AP, Porteintech, 1:1000).

### Statistical analysis

We expressed the data as representative images or the mean ± SD of each group from three independent experiments. We compared the data from multiple groups using ANOVA and post hoc Bonferroni analysis and the data from two groups by Student’s T-test using the SPSS version 18.0 (SPSS, Chicago, IL, USA). We analysed the potential correlation of two variables by Spearman’s rank test. We defined statistical significant difference when a *P*-value of < 0.05 and labelled the significant levels as *P < 0.05, **P < 0.01, ***P < 0.001 in the figures.

## Results

### TRPM7 silencing attenuates the growth of ovarian cancer by shifting glucose metabolic reprogramming

**Fig. 1:**
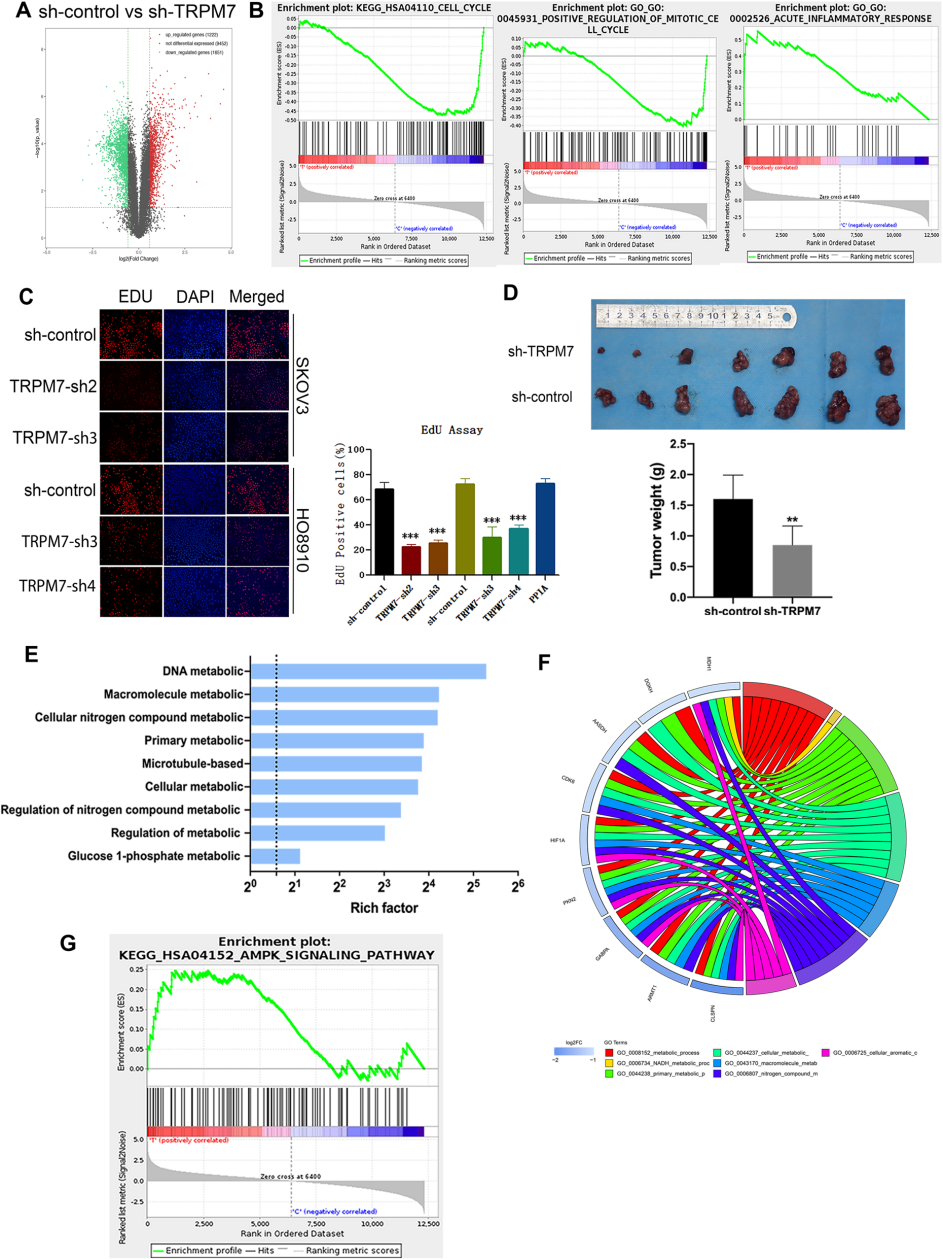
TRPM7 promotes the growth of ovarian cancer. **A** A volcano plot displayed the DEGs between sh-control SKOV3 and sh-TRPM7 SKOV3 cells. **B** GSEA analysis predicted that TRPM7 was involved in cell cycling and positive regulation of cell mitosis and acute inflammatory. **C** EdU assay exhibited that TRPM7 silencing inhibited cell proliferation. Scale bar = 50 μm. **D** TRPM7 silencing inhibited the growth of xenograft SKOV3 in vivo. **E** GO analysis exhibited that the DEGs were enriched in the indicated biological processes in the TRPM7 silencing SKOV3 cells. **F** Circos plot displayed the relationships between the DEGs and potential functions in TRPM7 silencing SKOV3 cells. (G) GSEA analysis predicted that TRPM7 was involved in the AMPK pathway

Given that up-regulated TRPM7 expression is related to poor prognosis of ovarian cancer [17], we determined the impact of TRPM7 expression on cell proliferation. First, we determined the expression of TRPM7 in the cell line and generated TRPM7 stably silencing SKOV3 and HO8910 cells, that results are shown in Supplementary Fig. 2 A-C. To understand how TRPM7 silencing regulated gene expression in ovarian cancer cells, we performed RNA-seq analyses between SKOV3 sh-Control and SKOV3 sh-TRPM7 cells. We found that with a fold change of ≥2.0 and *P* < 0.05, there were 2873 differentially expressed genes (DEGs) between SKOV3 sh-Control and SKOV3 sh-TRPM7 cells, and among the DEGs, 1222 were up-regulated and 1651 were down-regulated in SKOV3 sh-TRPM7 cells (Fig. 1A), which were supported by the Gene Set Enrichment Analysis (GSEA, Fig. 1B). An additional figure and table shows this in more detail [see Supplementary Table 4, and Supplementary Fig. 2 D and E] The GO and KEGG analyses indicated that the DEGs were involved in the process of cell cycling, positive regulation of mitotic cell cycling and acute inflammatory response in ovarian cancer (Table 1). To test the functional outcomes of TRPM7 silencing, we quantified the proliferation of different groups of ovarian cancer cells by the EdU assay. As shown in Fig. 1C, there were less numbers of EdU^+^ cells in the TRPM7-silenced SKOV3 or HO8910 cells than their sh-control cells. Furthermore, TRPM7 silencing significantly decreased the tumor sizes and weights in a mouse xenograft of SKOV3 tumors (*P* < 0.01, Fig. 1D). Given that rapid proliferation and metabolic reprogramming are hallmarks of cancer cells, we further performed the GO analyses and found that the DEGs were enriched in metabolic processes, such as cellular processes, DNA metabolism, macromolecule metabolism, primary metabolism, cellular metabolism, regulation of metabolism, and others (Fig. 1E). It is notable that many DEGs regulated glycolysis and OXPHOS, and they included the glycolysis-related PDK1, HK2, LDHB and OXPHOS-related IDH3B, UQCRC1, UQCRC2. TRPM7 silencing also down-regulated HIF-1α expression in SKOV3 cells (Fig. 1F). Further GSEA analyses revealed the potential signaling pathways, such as the AMPK signal pathway, were enriched in TRPM7-silenced SKOV3 cells (Fig. 1G, Table 2). Accordingly, we speculate that TRPM7 silencing may suppress the proliferation of ovarian cancer cells by shifting metabolic reprogramming.

**Table 1:** KEGG Pathways and GO annotation enriched in high-risk and low-risk groups using GSEA

NAME	SIZE	ES	NES	NOM ***p***-val	FDR q-val	FWER ***p***-val	Rank at max	Leading edge
KEGG_HSA04110_CELL_CYCLE	116	−0.4744268	−1.329925	0	0.32765228	1	2757	tags = 45%, list = 22%, signal = 57%
GO_GO:0045931_POSITIVE_REGULATION_OF_MITOTIC_CELL_CYCLE	121	−0.4059705	−1.7015916	0	0.2011301	0.758	1579	tags = 26%, list = 13%, signal = 29%
GO_GO:0002526_ACUTE_INFLAMMATORY_RESPONSE	32	0.55722696	1.6772016	0	0.41694513	0.837	895	tags = 25%, list = 7%, signal = 27%

**Table 2:** KEGG Pathways enriched in high-risk and low-risk groups using GSEA

NAME	SIZE	ES	NES	NOM ***p***-val	FDR q-val	FWER ***p***-val	Rank at max	Leading edge
KEGG_HSA04152_AMPK_SIGNALING_PATHWAY	95	0.2476375	1.2687452	0	0.4344668	1	1244	tags = 14%, list = 10%, signal = 15%

### TRPM7 silencing shifts metabolic reprogramming to OXPHOS in ovarian cancer cells

**Fig. 2:**
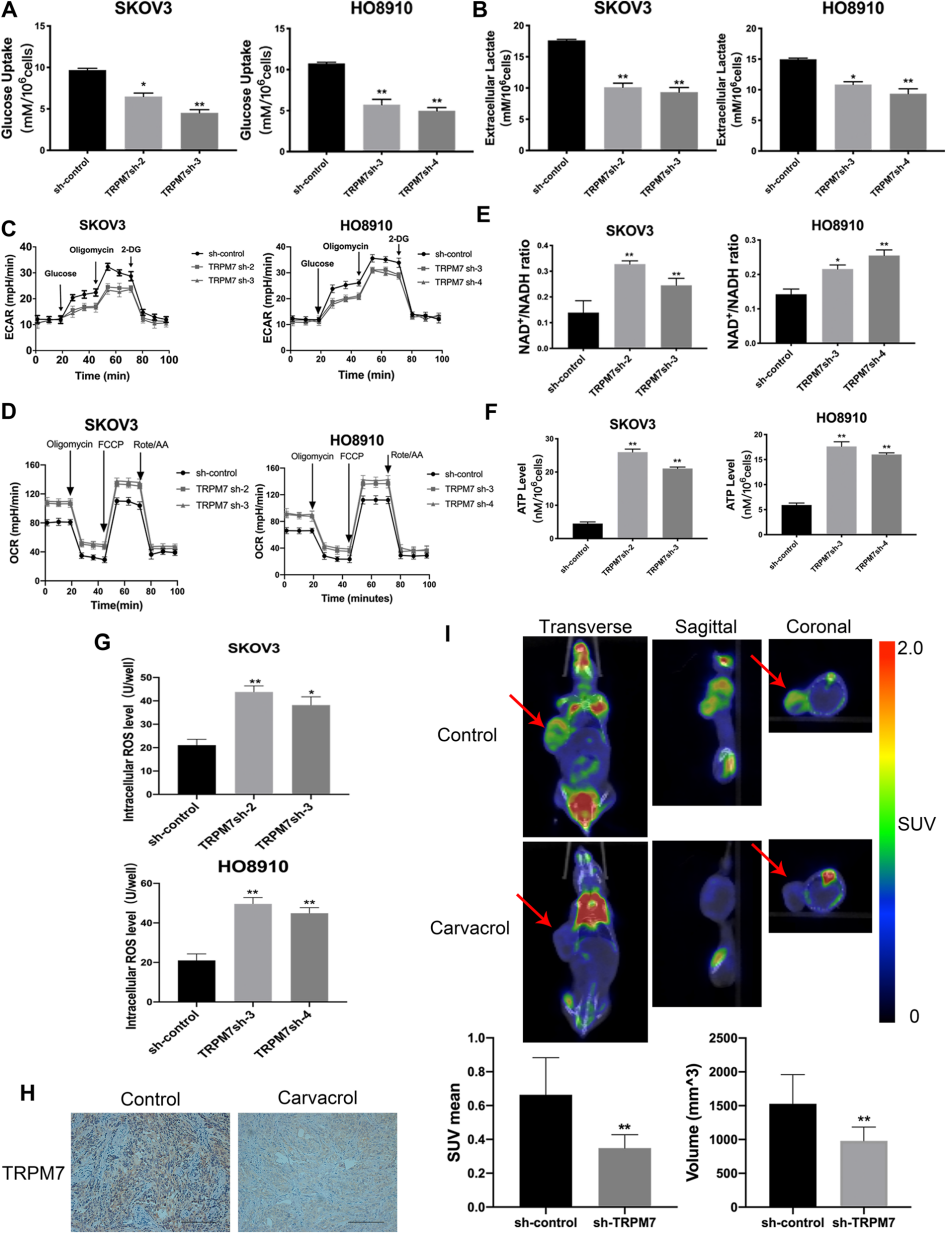
TRPM7 silencing shifts glycolysis to OXPHOS in ovarian cancer cells. **A** The effect of TRPM7 silencing on the glucose uptake levels, (**B**) extracellular lactate levels, (**C**) ECARs, (**D**) OCRs, (**E**) NAD^+^/NADH ratios, (**F**) ATP levels, (**G**) intracellular ROS levels in SKOV3 and HO8910 cells. **H** IHC analysis of TRPM7 expression in SKOV3 xenograft tumors. Scale bar = 50 μm. **I** Representative photographs of 18F-FDG PET/CT scans of SKOV3 xenograft tumors in mice following treatment with, or without, carvacrol, a potent inhibitor of TRPM7. SKOV3 and carvacrol were injected to right forelimbs. The SUV max was lower in the carvacrol group than in the control group (*n* = 7 per group)

Tumor cells usually prefer glycolysis to provide necessary energy and metabolites for their proliferation and switching to OXPHOS can promote high levels of ROS production [22]. Firstly, analysis of the basal glycolysis/OXPHOS ratio revealed that both SKOV3 and H808910 cells preferred glycolysis in our experimental conditions, and the result is shown in Supplementary Fig. 3A. To test whether TRPM7 silencing could shift the glucose metabolic reprogramming, we measured the levels of glucose consumption and lactic acid production in the sh-control and TRPM7 silencing ovarian cancer cells. In comparison with the sh-control cells, TRPM7 silencing significantly reduced the levels of glucose consumption by > 50% and lactic acid production by 35–40% in both SKOV3 and HO8910 cells (*P* < 0.05, *P* < 0.01, Fig. 2A and B). Seahorse assays exhibited that TRPM7 silencing also decreased ECRA, but increased OCR (Fig. 2C and D). TRPM7 silencing also increased the ratios of NAD^+^/NADPH and the levels of ATP and ROS in both SKOV3 and HO8910 cells (*P* < 0.05, *P* < 0.01, Fig. 2E-G). Immunohistochemistry (IHC) displayed that carvacrol treatment reduced the expression of TRPM7 in xenograft ovarian tumor tissues in mice in SKOV3 cells in vitro (Fig. 2H). In addition, the western blot experiment obtained the same result, and it is shown in Supplementary Fig. 3B. More importantly, PET-CT imaging revealed that carvacrol treatment not only reduced the tumor size, but also attenuated ^18^F-FDG uptake in tumor-bearing mice by significantly decreasing mean standard uptake value and total volume (*P* < 0.01, Fig. 2I). Collectively, these results indicate that TRPM7 silencing shifts glycolysis to OXPHOS in ovarian cancer, inhibiting ovarian cancer growth.

### TRPM7 silencing attenuates the glycolysis process and enhances the OXPHOS process in ovarian cancer cells

**Fig. 3:**
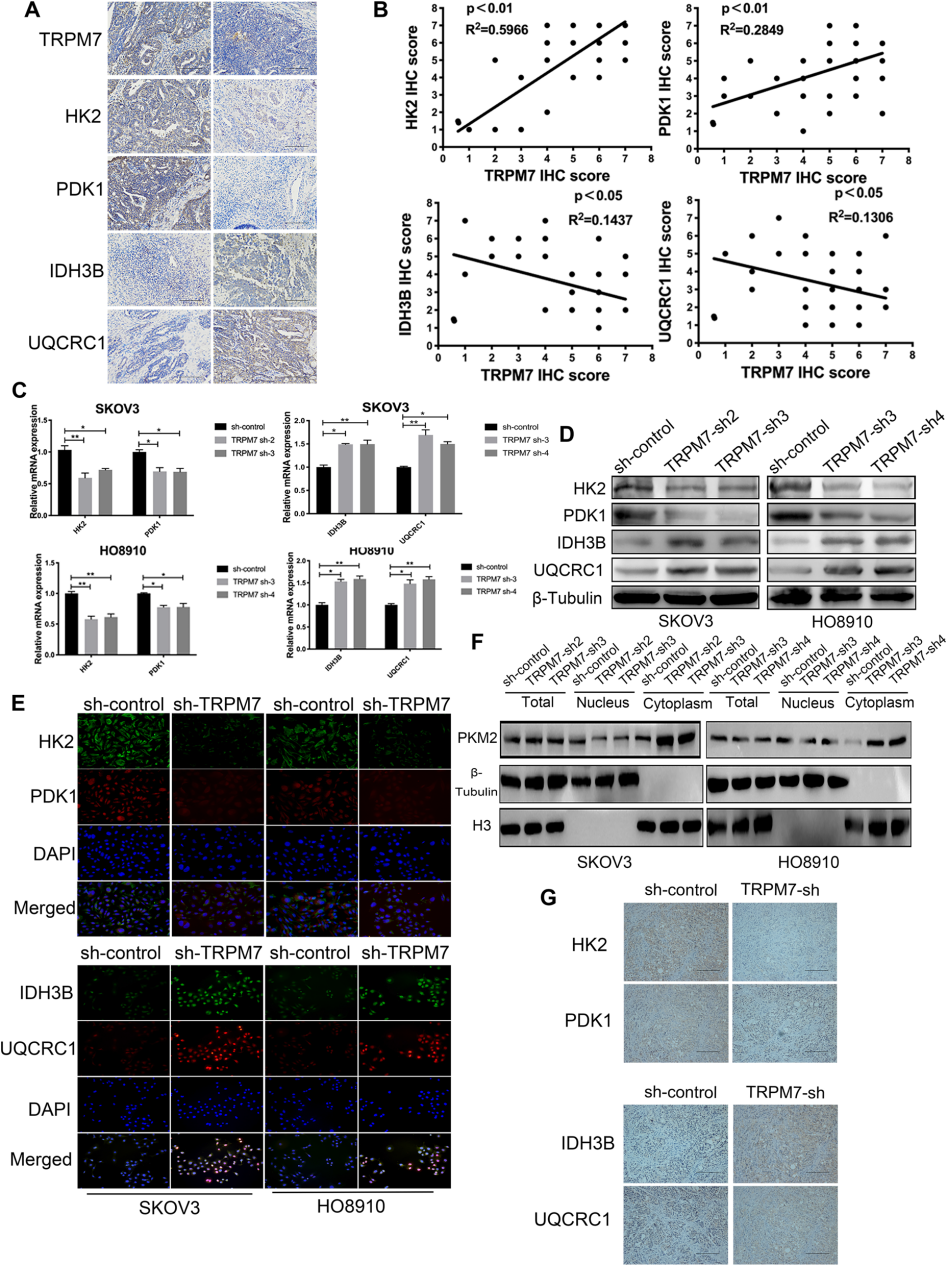
TRPM7 silencing reduces the expression of glycolysis-related regulators, but enhances the OXPHOS-related regulators in ovarian cancer cells. **A** IHC analysis of TRPM7, HK2, PDK1, IDH3B and UQCRC1 expression in human ovarian cancer tissues and non-tumor ovarian tissues. Scale bar = 50 μm. **B** Correlation analysis between the levels of TRPM7 and HK2, PDK1, IDH3B, or UQCRC1 expression in ovarian cancer tissues. **C-D** Western blot and RT-qPCR analyses of HK2, PDK1, IDH3B and UQCRC1 expression in the indicated cells. **E** Immunofluorescent analysis of HK2, PDK1, IDH3B and UQCRC1 expression in ovarian cancer cells after stained with mouse anti-HK2, rabbit anti-PDK1, mouse anti-IDH3B, rabbit anti-UQCRC1 and subsequent Alexa Fluor™488-goat anti-mouse IgG and Alexa Fluor™ 594-goat anti-rabbit IgG as well as DAPI, scale bar = 50 μm. **F** Western blot analysis of PKM2 in the nuclear and cytoplasmic fractions following TRPM7 silencing in SKOV3 and HO8910 cells. **G** IHC analysis of HK2, PDK1, IDH3B and UQCRC1 expression (scale bar = 50 μm)

To understand how TRPM7 modulated metabolic reprogramming in ovarian cancer, we analyzed the expression of TRPM7, glycolysis-related HK2, PDK1, and OXPHOS-related IDH3B and UQCRC1 in 60 ovarian cancer tissues by IHC (Fig. 3A). The information of those patients is shown in Supplementary Table 3. Correlation analysis indicated that TRPM7 expression levels in ovarian cancer tissues were positively correlated with HK2 (*P* < 0.01) and PDK1 (*P* < 0.01), but negatively correlated with IDH3B (*p* < 0.01) and UQCRC1 in this population (*P* < 0.05, Fig. 3B), supporting the notion that TRPM7 promotes glycolysis in ovarian cancer. Consequently, TRPM7 silencing decreased the relative HK2 and PDK1 mRNA transcripts, but increased IDH3B and UQCRC1 mRNA transcripts in both SKOV3 and HO8910 cells (*P* < 0.05, *P* < 0.01, Fig. 3C). Similar patterns of the relative levels of HK2, PDK1, IDH3B and UQCRC1 proteins were detected in these cells by Western blot and immunofluorescence (Fig. 3D and E), and the statistical analysis is shown in Supplementary Fig. 4A. PKM2 is a key enzyme for glycolysis and its nuclear translocation is associated with malignant behaviors of tumor cells [23]. To test the importance of PKM2, we characterized the intracellular distribution of PKM2 and found that TRPM7 silencing obviously decreased the levels of nuclear PKM2, but increased its cytoplasmic PKM2 in both SKOV3 and HO8910 cells (Fig. 3F). An additional figure shows statistical analysis in Supplementary Fig. 4B. Finally, decreased levels of HK2 and PDK1 proteins, but increased levels of IDH3B and UQCRC1 expression were observed in TRPM7-silenced SKOV3 tumors (Fig. 3G). Hence, TRPM7 silencing modulated the levels of glycolysis- and OXPHOS-related enzyme expression to shift glycolysis to OXPHOS in ovarian cancer.

### Activation of the AMPK signal pathway inhibits glycolysis that is dependent on high levels of HIF-1α in ovarian cancer cells

**Fig. 4:**
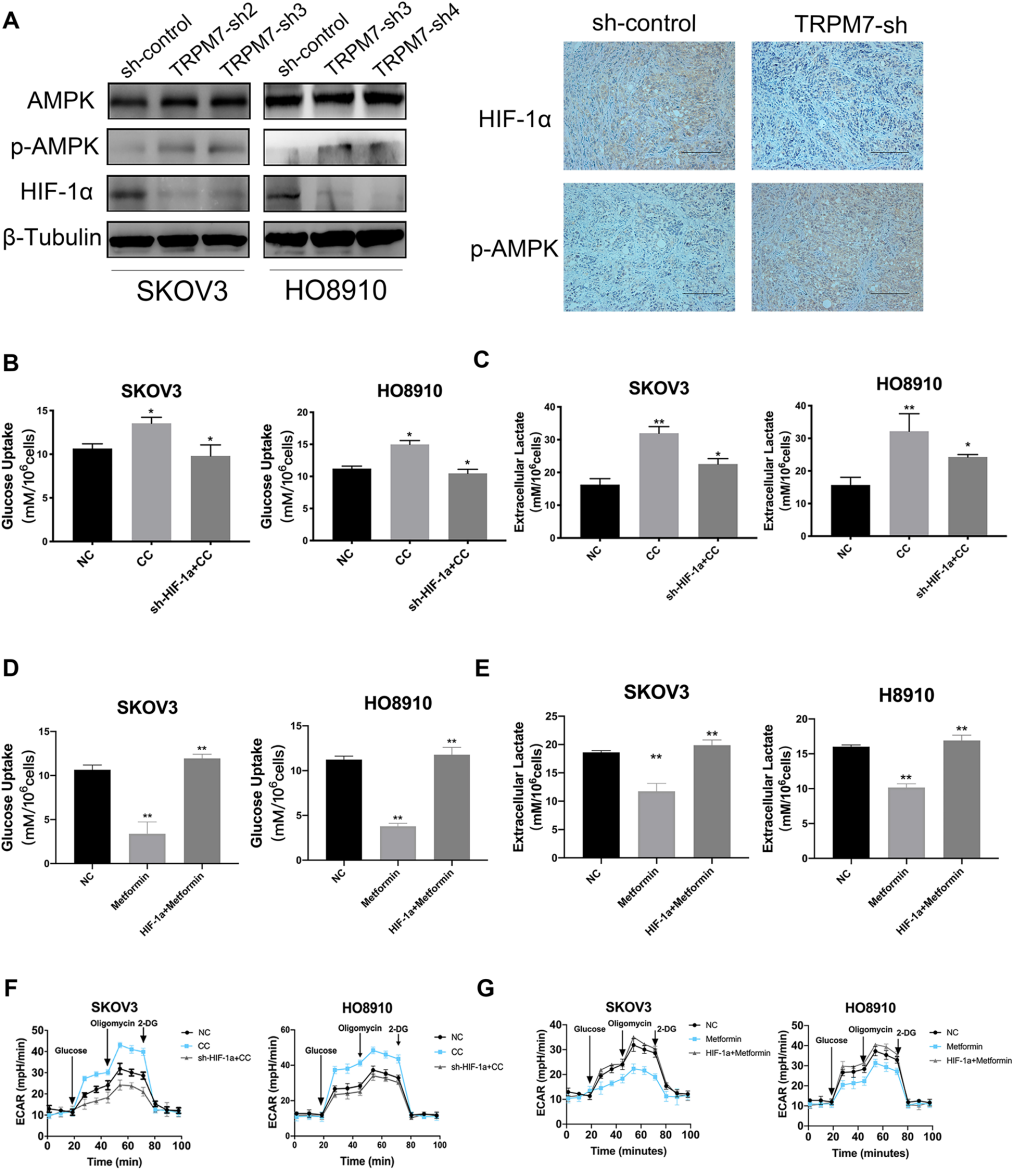
TRPM7 silencing enhances the AMPK activation, which is associated with inhibiting glycolysis in ovarian cancer cells. **A** Western blot and IHC analyses of HIF-1α expression and AMPK phosphorylation in the indicated cells and xenograft SKOV3 tumors (scale bar = 50 μm). **B**, **C**, **F** Treatment with CC to inhibit AMPK activation enhanced glycolysis, which was mitigated by HIF-1α silencing, by measuring glucose uptake (**B**), lactic acid production (**C**) and ECAR (**F**) in SKOV3-sh-control, SKOV3-sh-TRPM7, HO8910-sh-control and HO8910-sh-TRPM7 cells. **D**, **E**, **G** Treatment with metformin to enhance the AMPK activation attenuated glycolysis, which was abrogated by HIF-1α over-expression, by measuring glucose uptake (**D**), lactic acid production (**E**) and ECAR (**G**) in the indicated cells

It is well known that AMPK activation can promote OXPHOS and inhibit glycolysis by down-regulating the function of HIF-1α that can promote glycolysis in tumor cells under hypoxic conditions [24–27]. To understand how TRPM7 silencing modulates the HIF-1α and AMPK signaling, we characterized the levels of HIF-1α and AMPK expression and activation in the indicated ovarian cancer cells by Western blot and IHC. Clearly, TRPM7 silencing elevated the relative levels of AMPK phosphorylation, but minimized the HIF-1α expression in SKOV3 and HO8910 cells and SKOV3 tumors (Fig. 4A). An additional figure shows the statistical analysis in Supplementary Fig. 4C. Hence, TRPM7 silencing enhanced AMPK activation and reduced HIF-1α protein to switch preferable glycolysis to OXPHOS in ovarian cancer cells. To further understand the importance of AMPK activation in shifting glycolysis to OXPHOS, we tested the effect of altered AMPK activation on the HIF-1α-regulated glycolysis in ovarian cancer cells. We first established stable HIF-1α silencing SKOV3 and HO8910 cells. Compared with the vehicle-treated control cells, treatment with Compound C (CC, an inhibitor of AMPK) significantly inhibited AMPK activation (the consequences are shown in Supplementary Fig. 5A and B), but increased glucose uptake, lactate acid production and ECAR in both SKOV3 and HO8910 cells (Fig. 4B, C and F), which were significantly mitigated or abrogated in the HIF-1α silencing SKOV3 and HO8910 cells. In contrast, treatment with metformin (an activator of AMPK) not only increased AMPK phosphorylation (the results are shown in Supplementary Fig. 5C and D), but also significantly decreased glucose uptake, lactic acid production and ECAR in both SKOV3 and HO8910 cells (Fig. 4D, E and G). However, the decreased glycolysis by metformin was abrogated by HIF-1α over-expression in ovarian cancer cells. These two lines of evidence demonstrated that AMPK activation inhibited glycolysis that was dependent on high levels of HIF-1α presence in ovarian cancer.

### AMPK activation or HIF-1α over-expression mitigates the TRPM7 silencing-inhibited glycolysis in ovarian cancer cells

**Fig. 5:**
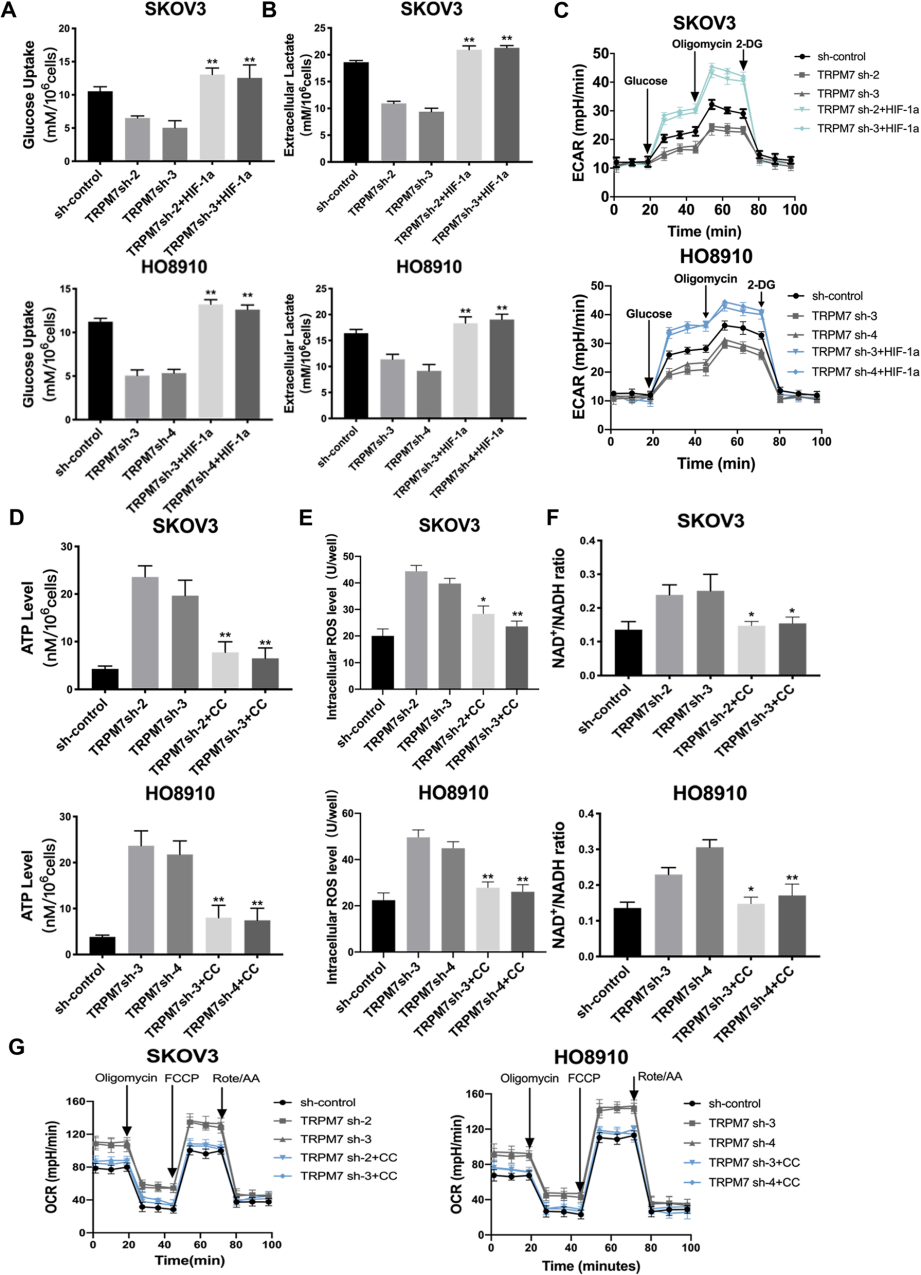
The shifting glycolysis to OXPHOS by TRPM7 silencing is abrogated or mitigated by HIF-1α over-expression or AMPK inhibition in ovarian cancer cells. HIF-1α over-expression abrogated the TRPM7 silencing-promoted glycolysis in ovarian cancer cells, determined by the glucose uptake (**A**), lactic acid production (**B**) and ECAR (**C**). Treatment with CC (20 μM) to inhibit AMPK activation mitigated the TRPM7 silencing-induced OXPHOS in ovarian cancer cells, determined by the ATP levels (**D**), ROS levels (**E**), NAD^+^/NAPH ratios (**F**) and OCR (**G**)

To understand how HIF-1α and AMPK activation regulate the TRPM7 silencing-induced metabolic reprogramming in ovarian cancer cells, we first generated HIF-1α over-expression in TRPM7 silencing ovarian cancer cells. Second, we tested the impact of TRPM7 silencing on the HIF-1α-mediated glycolysis in SKOV3 and HO8910 cells. Comparison with the control cells, TRPM7 silencing significantly decreased glucose uptake, lactic acid production and ECAR in SKOV3 and HO8910 cells, but did not significantly change glucose uptake, lactic acid production and ECAR in the HIF-1α over-expressing SKOV3 and HO8910 cells (Fig. 5A-C). These data indicated that TRPM7 deficiency suppressed glycolysis in ovarian cancer cells, which was abrogated by HIF-1α over-expression. Next, we tested how inhibition of AMPK modulated the TRPM7 silencing-promoted OXPHOS in ovarian cancer cells. While TRPM7 silencing significantly increased the production of ATP, ROS and the ratios of NAD^+^/NADPH as well as OCR in ovarian cancer cells, which were significantly mitigated or abrogated by treatment with CC (Fig. 5D-G). Thus, HIF-1α over-expression significantly mitigated or abrogated the TRMP7 silencing-decreased glycolysis and inhibition of AMPK abrogated the TRPM7 silencing-enhanced OXPHOS in ovarian cancer cells.

### TRPM7 silencing enhances the AMPK activation and decreases HIF-1α protein levels to shift glycolysis to OXPHOS in ovarian cancer cells

**Fig. 6:**
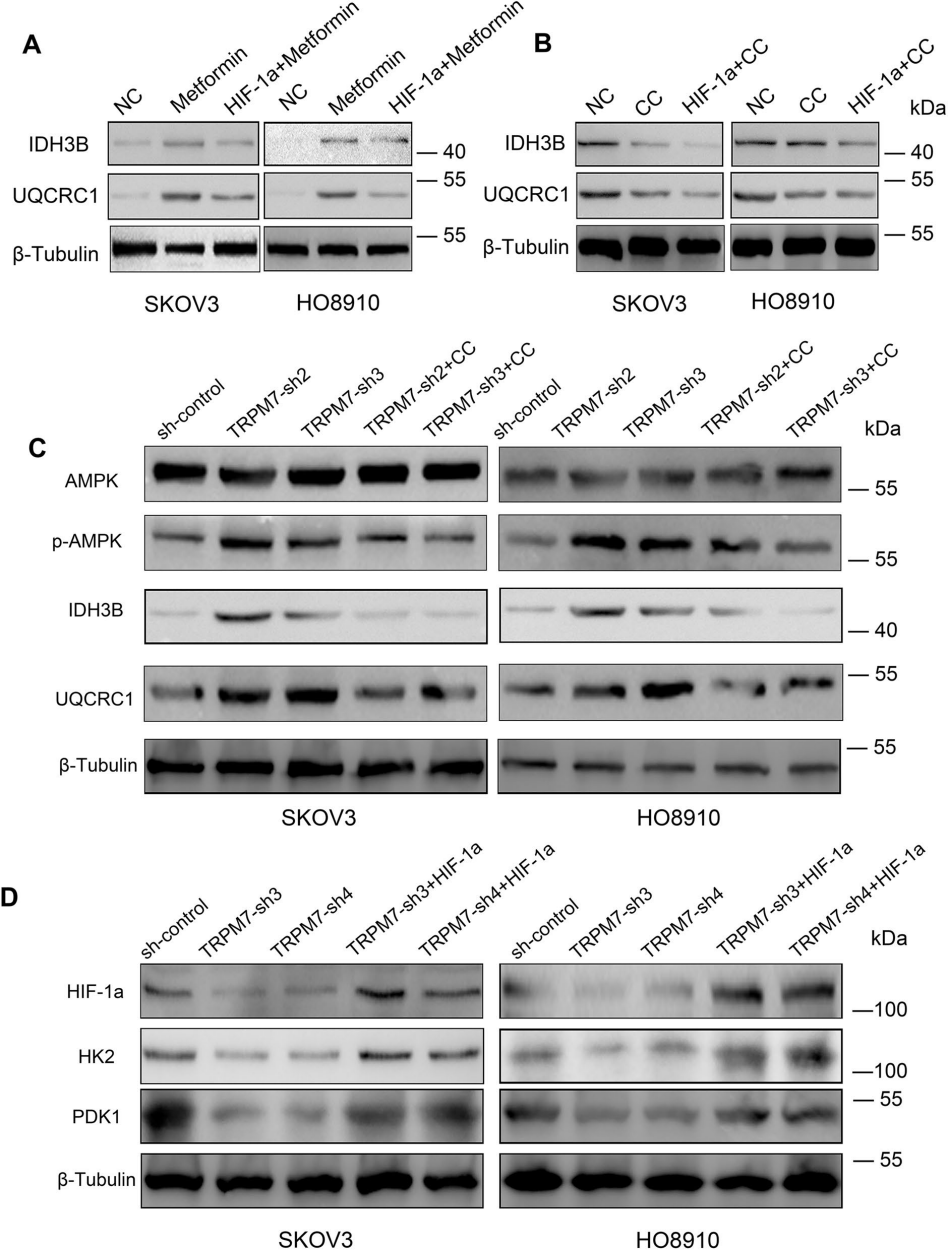
Altered AMPK activation or HIF-1α expression modulates the TRPM7 silencing-decreased expression of glycolysis-relative regulators and increased expression of OXPHOS-relative regulators in ovarian cancer cells. **A-D** Western blot analysis of IDH3B, UQCRC1, HIF-1α, HK2, PDK1 expression and AMPK phosphorylation in the indicated groups of cells

Next, we explored how TRPM7 silencing modulated AMPK activation, HIF-1α expression, OXPHOS and glycolysis in ovarian cancer cells. We initially quantified the IDH3B and UQCRC1 expression in the different groups of cells. Relative to the untreated control cells, treatment with metformin to activate AMPK obviously increased IDH3B and UQCRC1 expression, which were mitigated by HIF-1α silencing in SKOV3 and HO8910 cells (Fig. 6A). In contrast, treatment with CC to inhibit AMPK activation remarkably decreased the relative levels of IDH3B and UQCRC1 expression, which were further reduced in the HIF-1α silencing SKOV3 and HO8910 cells (Fig. 6B). Similarly, TRPM7 silencing increased AMPK phosphorylation, IDH3B and UQCRC1 expression in those cells, which were limited by CC (Fig. 6C). In contrast, TRPM7 silencing reduced the HIF-1α, HK2 and PDK1 expression in SKOV3 and HO8910 cells, which were rescued by induction of HIF-1α over-expression (Fig. 6D). An additional figure shows Statistical Analysis of Western blot in Supplementary Fig. 6. Together, TRPM7 silencing enhances the AMPK activation and decreases HIF-1α protein levels to shift glycolysis to OXPHOS in ovarian cancer cells.

### TRPM7 silencing enhances the AMPK activation to promote HIF-1α ubiquitination and degradation in ovarian cancer cells

**Fig. 7:**
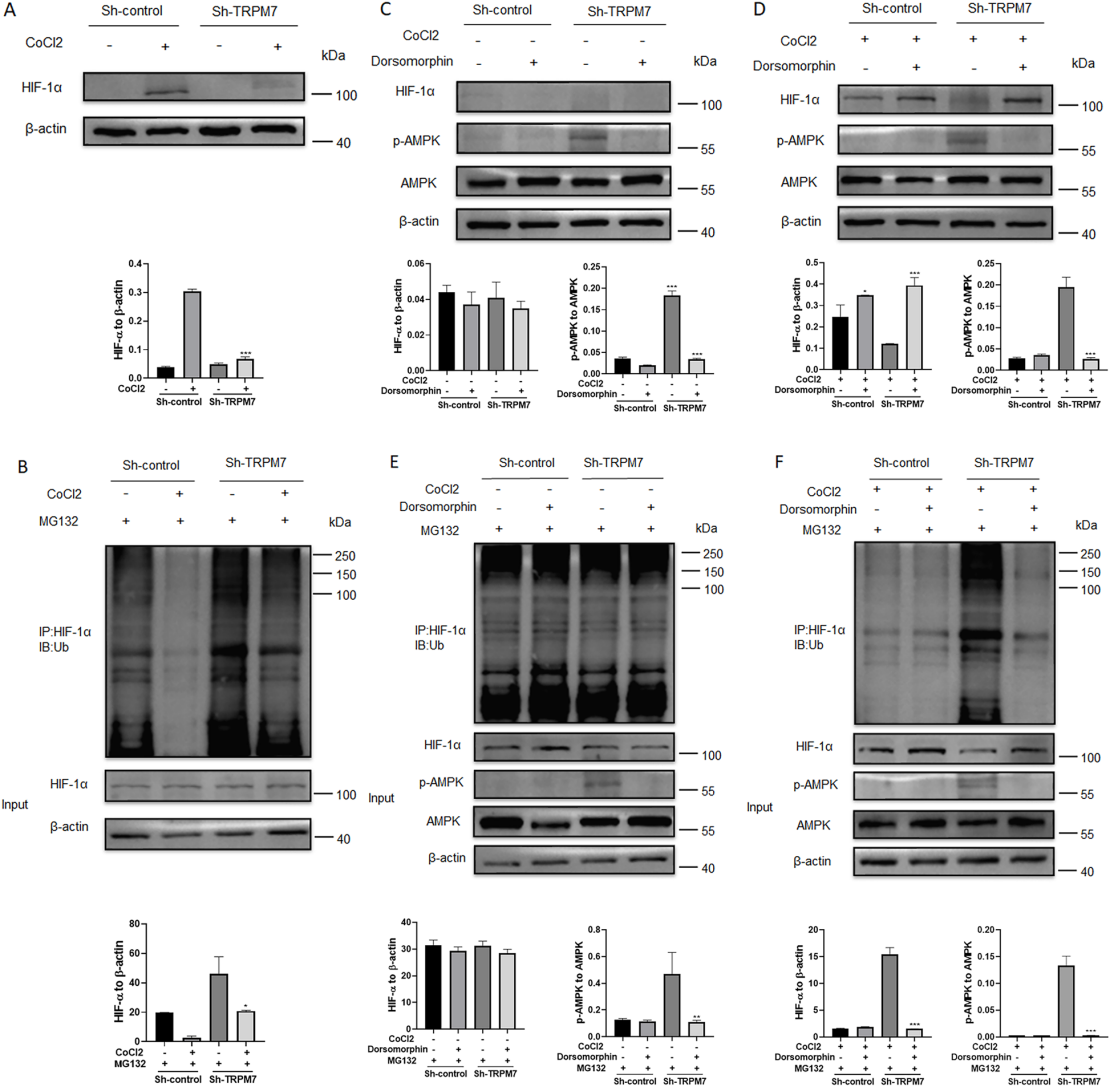
TRPM7 silencing activates the AMPK signaling to promote ubiquitination and degradation of HIF-1α in ovarian cancer cells. **A** Western blot displayed that TRPM7 silencing decreased HIF-1 protein levels in ovarian cancer cells under a hypoxic condition. **B** In vivo ubiquitination assays indicated that the decreased HIF-1α protein by TRPM7 silencing was abrogated by treatment with MG132, suggesting that TRPM7 silencing promoted HIF-1α ubiquitination and degradation in SKOV3 cells. **C** There was no detectable HIF-1α protein in the SKOV3 cells under a normoxic condition. **D** Inhibition of AMPK activation by CC increased HIF-1α protein levels in both control and TRPM7 silencing SKOV3 cells under a hypoxic condition. **E**, **F** In vivo ubiquitination assays revealed that treatment with CC to inhibit AMPK activation mitigated the TRPM7 silencing-induced HIF-1α ubiquitination and degradation in ovarian cancer cells under both normoxic and hypoxic conditions

Finally, we investigated how TRPM7 silencing decreased HIF-1α protein levels in ovarian cancer cells. Hypoxia induced HIF-1α protein expression in ovarian cancer cells, which was very weak in the TRPM7 silencing ovarian cancer cells (Fig. 7A). Treatment with MG132, a proteasome inhibitor, remarkably restored HIF-1α protein levels in ovarian cancer cells regardless of hypoxia condition and TRPM7 silencing (Fig. 7B), indicating that TRPM7 silencing promoted the ubiquitination and proteasomal degradation of HIF-1α. Furthermore, treatment with CC to inhibit AMPK activation restored HIF-1α protein levels in both control and TRPM7 silencing ovarian cancer cells under hypoxic and normoxic conditions (Fig. 7C and D). Moreover, treatment with CC also decreased the ubiquitination of HIF-1α in both control and TRPM7 silencing ovarian cancer cells under hypoxic and normoxic conditions (Fig. 7E and F). Therefore, TRPM7 silencing enhanced AMPK activation to promote the ubiquitination and proteasomal degradation of HIF-1α, shifting glycolysis to OXPHOX in ovarian cancer cells.

## Discussion

Exploring the potential mechanisms for ovarian cancer growth and finding new therapeutic targets have become a current research hotspot [28]. TRPM7 expression is up-regulated in various tumors [19, 29, 30]. Furthermore, up-regulated TRPM7 expression is positively related to pelvic lymph node metastasis and poor prognosis of ovarian cancer [31]. Inhibition of TRPM7 can minimize the malignant behaviors of ovarian cancer [17, 18]. Here, we reported that TRPM7 silencing changed the transcriptomes involved in modulating cell cycle and acute inflammation, which were linked to tumor cell proliferation [32–34] and modulated the tumor environment [35–38]. Actually, we found that TRMP7 silencing suppressed ovarian cancer cell proliferation and xenograft tumor growth in mice. Hence, TRPM7 acts as an oncogenic factor to promote ovarian cancer growth and is a potential target for development of therapies for ovarian cancer.

It is well known that glucose metabolism by the Warburg effect and OXPHOS can provide nutrients and energy to support the proliferation and biosynthesis in tumor cells [8, 39, 40]. Our results indicated that TRPM7 silencing shifted glycolysis to OXPHOS in ovarian cancer cells. Evidently, TRPM7 silencing significantly minimized glucose uptake, lactic acid production, ECAR, but elevated ATP and ROS levels, NAD^+^/NADH ratios and OCR in ovarian cancer cells. Consistently, PET-CT imaging revealed that treatment with carvacrol to inhibit TRPM7 activity also decreased 18F-FDG uptake by xenograft ovarian cancer in mice. Furthermore, TRPM7 silencing also decreased the relative levels of glycolysis-related HK2 and PDK1 expression as well as PKM2 nuclear translocation, but increased OXPHOS-related IDH3B and UQCRC1 expression in ovarian cancer cells and tissues. The PKM2 is a specific isoform of pyruvate kinase and a rate-limiting enzyme controlling the glycolysis process. The PKM2 is highly expressed in many types of tumors, including ovarian cancer [41–45]. Studies have shown that PKM2 can translocate to the nucleus, where it forms a complex with HIF-1α and acts as a transcription coactivator of HIF-1α to promote the transcription of HIF-1α targeted genes (such as glycolytic rate-limiting enzymes). Hence, TRPM7 silencing inhibited the proliferation of ovarian cancer cells by shifting from glycolysis to OXPHOS [46–48]. OXPHOS can produce high levels of ATP and ROS, which can induce oxidative inflammation and excessive ROS levels can damage DNA and cells, impairing their proliferation [35, 38, 49]. These may partially explain why TRPM7 silencing inhibited ovarian cancer cell proliferation and tumor growth.

**Fig. 8:**
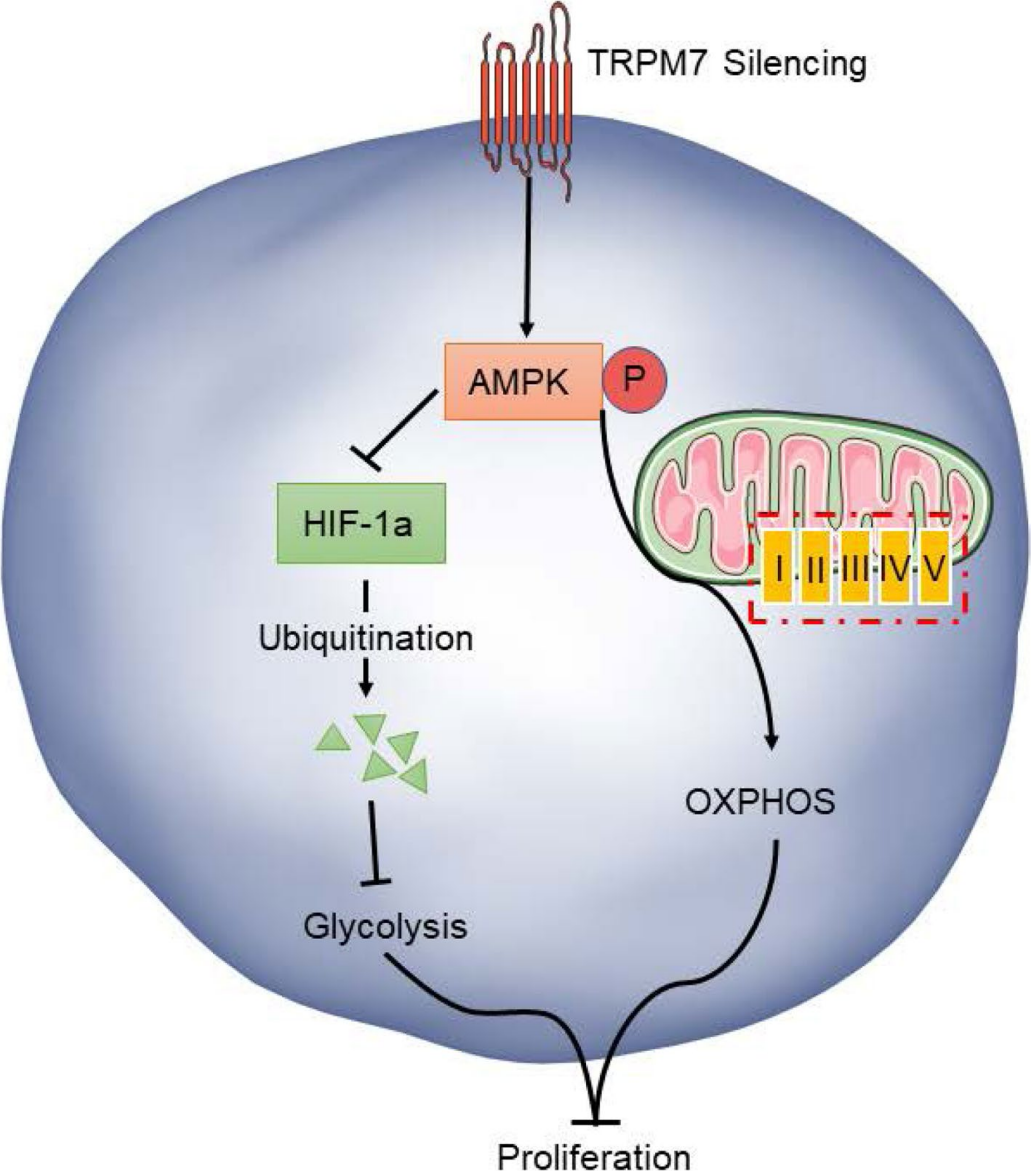
TRPM7 silencing shifts glycolysis to OXPHOS by modulating the AMPK / HIF-1α signaling in ovarian cancer cells. TRPM7 silencing can enhance the AMPK activation promote HIF-1α ubiquitination proteasomal degradation that attenuates the HIF-1α-enhanced glycolysis to shift glycolysis to OXPHOS, inhibiting ovarian cancer cell proliferation and tumor growth

Glucose metabolic reprogramming by converting glycolysis to OXPHOS or tumor cell metabolic plasticity has been noticed in several types of tumor cells and is regulated by the HIF-1α and AMPK signaling [9, 10, 26, 27]. Actually, the enhanced AMPK activation inhibits the expression of HIF-1α and glycolysis-related regulators of HK2, PKM, glucose transporter (GLUT1), fructose-6-phosphate-2-kinase / fructose-2,6-bisphosphatase 4 (PFKFB4) and lactate dehydrogenase A (LDHA) [10, 50–52]. Our data revealed that TRPM7 silencing down-regulated HIF-1α expression, but enhanced AMPK activation in ovarian cancer cells, extending previous observations [53–56]. Furthermore, treatment with metformin to enhance AMPK activation promoted the shifting from glycolysis to OXPHOS in ovarian cancer cells, which was abrogated by HIF-1α over-expression. This shifting may supply fewer nucleotides and lipid precursors for macromolecular synthesis in ovarian cancer cells. These, together with high levels of ROS to damage ovarian cancer cells, inhibit ovarian cancer growth. In contrast, treatment with CC to inhibit AMPK activation minimized the shifting from glycolysis to OXPHOS, dependent on high HIF-1α levels in ovarian cancer cells [53, 54, 57, 58]. Apparently, the HIF-1α and AMPK signaling has opposite roles in regulating glycolysis and OXPHOS in ovarian cancer. More importantly, we found that TRPM7 silencing promoted HIF-1α ubiquitination and degradation in ovarian cancer cells. It is possible that TRPM7 silencing may activate the AMPK to promote HIF-1α ubiquitination proteasomal degradation that attenuates the HIF-1α-enhanced glycolysis to shift glycolysis to OXPHOS, inhibiting the proliferation of ovarian cancer cells (Fig. 8). Conceivably, these findings may uncover the regulation of the TRPM7/AMPK/HIF-1α axis on the glucose metabolic reprogramming in ovarian cancer.

## Conclusion

Our data indicated that TRPM7 silencing shifted glycolysis to OXPHOS in ovarian cancer cells and attenuated ovarian cancer cell proliferation and tumor growth. Mechanistically, TRPM7 silencing enhanced AMPK activation and promoted HIF-1α ubiquitination and degradation in ovarian cancer cells. Hence, our findings may uncover the new function of TRPM7 in the growth of ovarian cancer and imply that TRPM7 may be a new target for design of therapies for ovarian cancer.

## Supplementary Information

**Additional file 1: Supplementary Table 1**. The target sequences of sh-TRPM7

**Additional file 2: Supplementary Table 2**. The sequences of primers

**Additional file 3: Supplementary Table 3**. The characteristics of the patients (*n* = 60)

**Additional file 4.**

**Additional file 5: Supplementary Fig. 1.** The animal experimental models and PET/CT study.

**Additional file 6: Supplementary Fig. 2**. Verification of TRPM7 silencing in ovarian cancer cells. (A) Western blot analyses of TRPM7 expression in SKOV3 and HO8910 cells. SKOV3 and HO8910 cells were transduced with lentiviruses for expressing scramble RNA, TRPM7-sh1, TRPM7-sh2, TRPM7-sh3 or TRPM7-sh4, respectively. Four days later, the relative levels of TRPM7 expression in each group were quantified by RT-qPCR (B) and Western blot (C). (D) PCA score plot. (E) Heatmap displayed the DEGs between control and TRPM7 silencing SKOV3 cells.

**Additional file 7: Supplementary Fig. 3.** (A) ECARs and OCRs in SKOV3 and HO8910 cells. (B) Western blot analyses of TRPM7 expression in SKOV3 and HO8910 cells. (C) Western blot analysis of TRPM7 expression in SKOV3 cells following treatment with, or without, carvacrol.

**Additional file 8: Supplementary Fig. 4.** The quantitative analysis of Western blot data in Fig. 3.

**Additional file 9: Supplementary Fig. 5**. Verification of HIF-1α silencing and over-expression as well as modulating AMPK activation in ovarian cancer cells by Western blot. (A) Western blot analysis of HIF-1α silencing in SKOV3 and HO8910 cells. (B) Treatment with CC attenuated AMPK activation in SKOV3 and HO8910 cells. (C) Western blot analysis of HIF-1α over-expression in SKOV3 and HO8910 cells. (D) Treatment with metformin enhanced AMPK activation in SKOV3 and HO8910 cells.

**Additional file 10: Supplementary Fig. 6.** The quantitative analysis of Western blot data in Fig. 6.

## Data Availability

The datasets supporting the conclusions of this article are included within the article.

## Abbreviations

TRPM7: Transient receptor potential 7
ECAR: Extracellular acidification rate
OCR: Oxygen consumption rate
OXPHOS: Oxidative phosphorylation
HIF: Hypoxia inducible factor
AMP: Adenosine 5′-monophosphate
AMPK: AMP-activated protein kinase
GSEA: Gene Set Enrichment Analysis
FDR: False discovery rate
GO: Gene Ontology
RT-qPCR: Quantitative real-time PCR
SDS-PAGE: Sodium dodecyl sulfate-polyacrylamide gel electrophoresis
PVDF: Polyvinylidene difluoride
DEGs: Differentially expressed genes
IHC: Immunohistochemistry
CC: Compound C
