# Supplementary material for: TRPM7 silencing modulates glucose metabolic reprogramming to inhibit the growth of ovarian cancer by enhancing AMPK activation to promote HIF-1α degradation
Source: J Exp Clin Cancer Res. 2022 Jan 31;41:44. doi: 10.1186/s13046-022-02252-1 (PMC8802454; doi:10.1186/s13046-022-02252-1)
Supplement: Supplementary file 1 — Additional file 1: Supplementary Table 1. The target sequences of sh-TRPM7 [file 13046_2022_2252_MOESM1_ESM.docx]

### Supplementary Table 1. The target sequences of sh-TRPM7

| **Target Sequences Name** | **Sequences** |
| --- | --- |
| TRPM7-shRNA1 | 5’-GGTGTTCCCAGAAAGGCAA-3’ |
| TRPM7-shRNA2 | 5’-AACCGGAGGTCAGGTCGAAAT-3’ |
| TRPM7-shRNA3 | 5’-AAGCAGAGTGACCTGGTAGAT-3’ |
| TRPM7-shRNA4 | 5’-CAATATGTTCTACATTGTA-3’ |
