# Supplementary material for: TRPM7 silencing modulates glucose metabolic reprogramming to inhibit the growth of ovarian cancer by enhancing AMPK activation to promote HIF-1α degradation
Source: J Exp Clin Cancer Res. 2022 Jan 31;41:44. doi: 10.1186/s13046-022-02252-1 (PMC8802454; doi:10.1186/s13046-022-02252-1)
Supplement: Supplementary file 2 — Additional file 2: Supplementary Table 2. The sequences of primers [file 13046_2022_2252_MOESM2_ESM.docx]

### Supplementary Table 2. The sequences of primers

| **Primer name** | **Primer sequences** |
| --- | --- |
| TRPM7 | forward 5'- TCCCGATAGATGGCTACAGG-3'  reverse 5'- CCTGACGAGTTGCTGACAAA-3' |
| HK2 | forward 5'-GACCAACTTCCGTGTGCTTT-3'  reverse 5'-TCCATGAAGTTAGCCAGGCA-3' |
| PDK1 | forward 5'-AGTTCATGTCACGCTGGGTA-3'  reverse 5'-CAGCTTCAGGTCTCCTTGGA-3' |
| IDH3B | forward 5'-ATGCTGCGGCATCTTAATCT-3'  reverse 5'-GCAATGACAGCCTCAGTGAA-3' |
| UQCRC1 | forward 5'- GGGCAAAAACATCCTCAGAA-3'  reverse 5'- ACGGATCCGGTTGTAGTCTG-3' |
| β-Tubulin | forward 5'- CTCTGAAGCTGACCACACCA-3'  reverse 5'- GCCAGGCATAAAGAAATGGA-3' |
