# Supplementary material for: TRPM7 silencing modulates glucose metabolic reprogramming to inhibit the growth of ovarian cancer by enhancing AMPK activation to promote HIF-1α degradation
Source: J Exp Clin Cancer Res. 2022 Jan 31;41:44. doi: 10.1186/s13046-022-02252-1 (PMC8802454; doi:10.1186/s13046-022-02252-1)
Supplement: Supplementary file 3 — Additional file 3: Supplementary Table 3. The characteristics of the patients (n = 60) [file 13046_2022_2252_MOESM3_ESM.docx]

### Supplementary Table 3. The characteristics of the patients (n=60)

| Characteristics | No. of patients |
| --- | --- |
| Ages  ≤60  >60 | 24  36 |
| Histologic type  Serous  Mucinous  Endometrioid | 53  5  2 |
| Pathological grade  1  2  3 | 14  20  26 |
| FIGO stage  I-II  III-IV | 31  29 |
| Pelvic metastasis  YES  NO | 43  17 |

Abbreviation, FIGO, International Federation of Gynecology and Obstetrics
