# Supplementary material for: TRPM7 silencing modulates glucose metabolic reprogramming to inhibit the growth of ovarian cancer by enhancing AMPK activation to promote HIF-1α degradation
Source: J Exp Clin Cancer Res. 2022 Jan 31;41:44. doi: 10.1186/s13046-022-02252-1 (PMC8802454; doi:10.1186/s13046-022-02252-1)
Supplement: Supplementary file 5 — Additional file 5: Supplementary Fig. 1. The animal experimental models and PET/CT study. [file 13046_2022_2252_MOESM5_ESM.pdf]

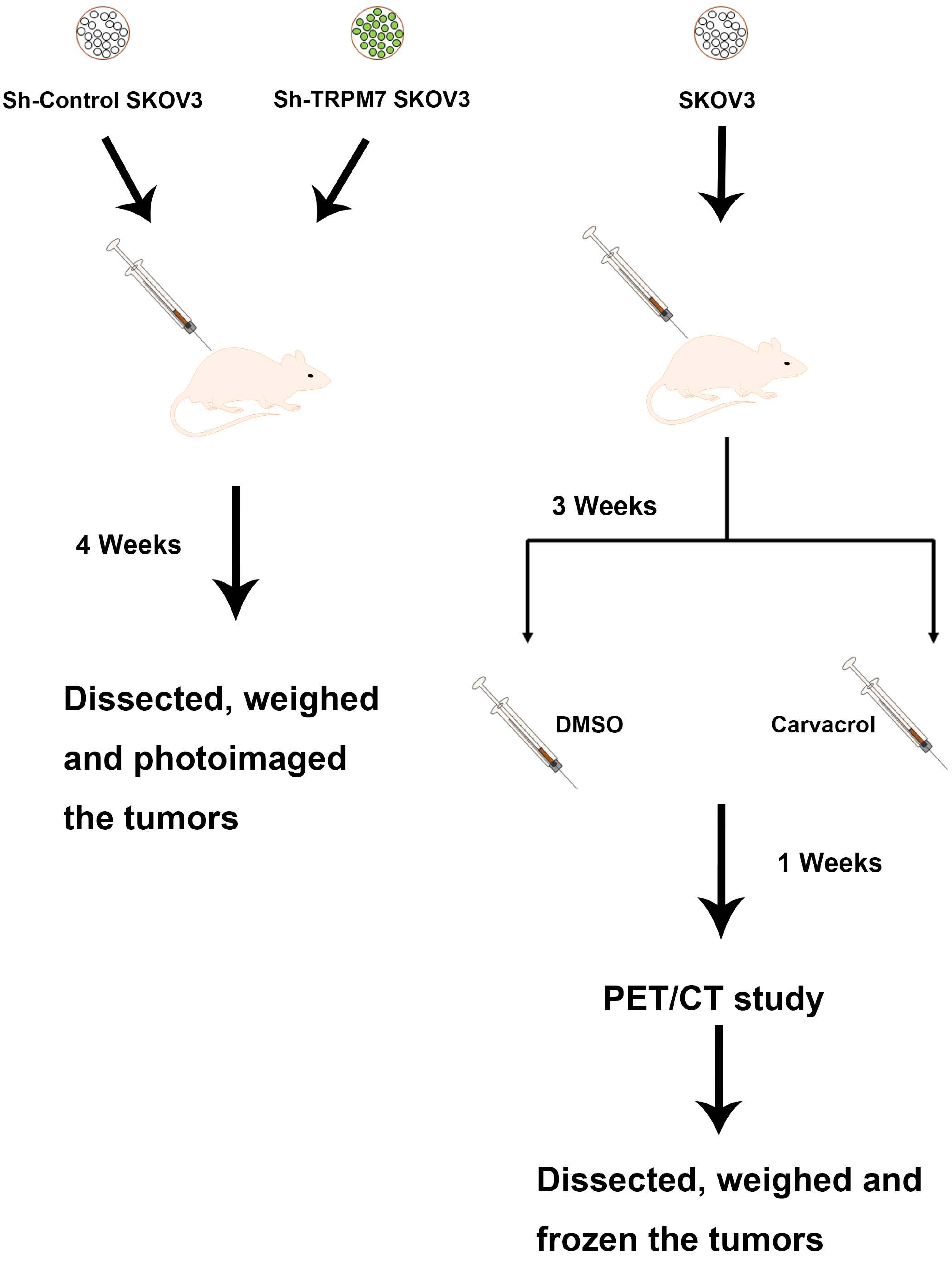
Sh-Control SKOV3
Sh-TRPM7 SKOV3
SKOV3
4 Weeks
Dissected, weighed and photoimaged the tumors
3 Weeks
DMSO
Carvacrol
1 Weeks
PET/CT study
Dissected, weighed and frozen the tumors
