# Supplementary figures and images for: TRPM7 silencing modulates glucose metabolic reprogramming to inhibit the growth of ovarian cancer by enhancing AMPK activation to promote HIF-1α degradation
Source: J Exp Clin Cancer Res. 2022 Jan 31;41:44. doi: 10.1186/s13046-022-02252-1 (PMC8802454; doi:10.1186/s13046-022-02252-1)

**A**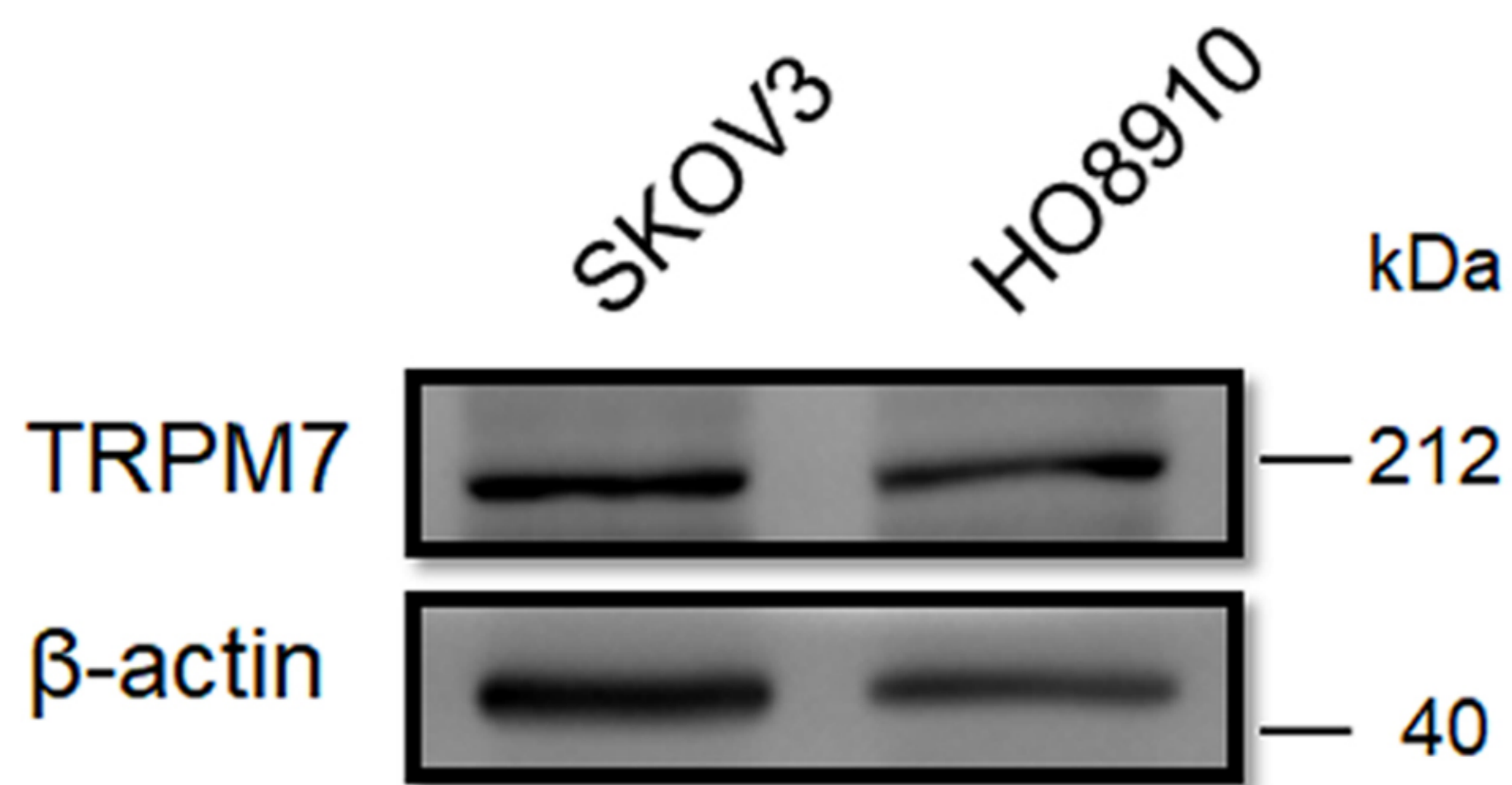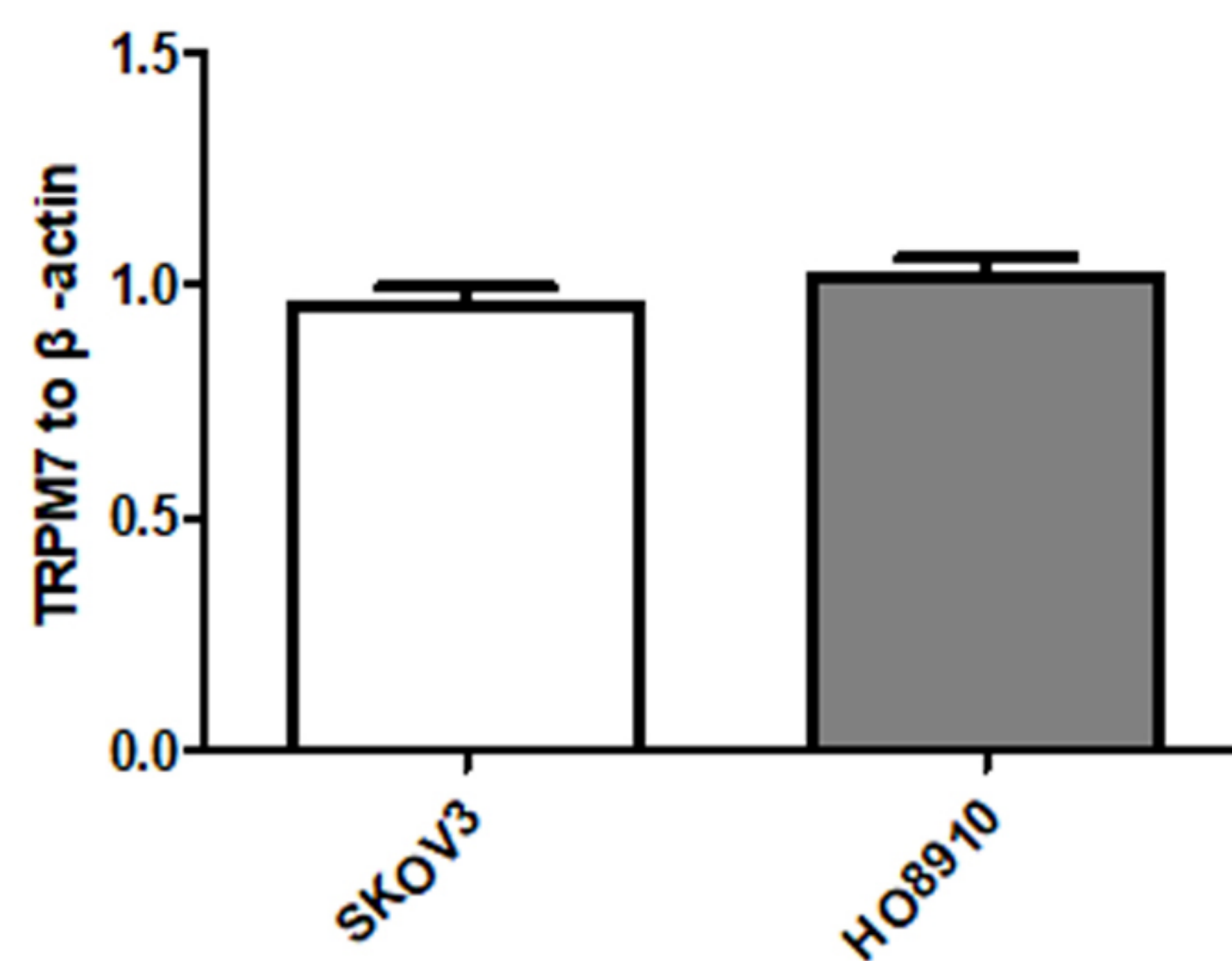**B**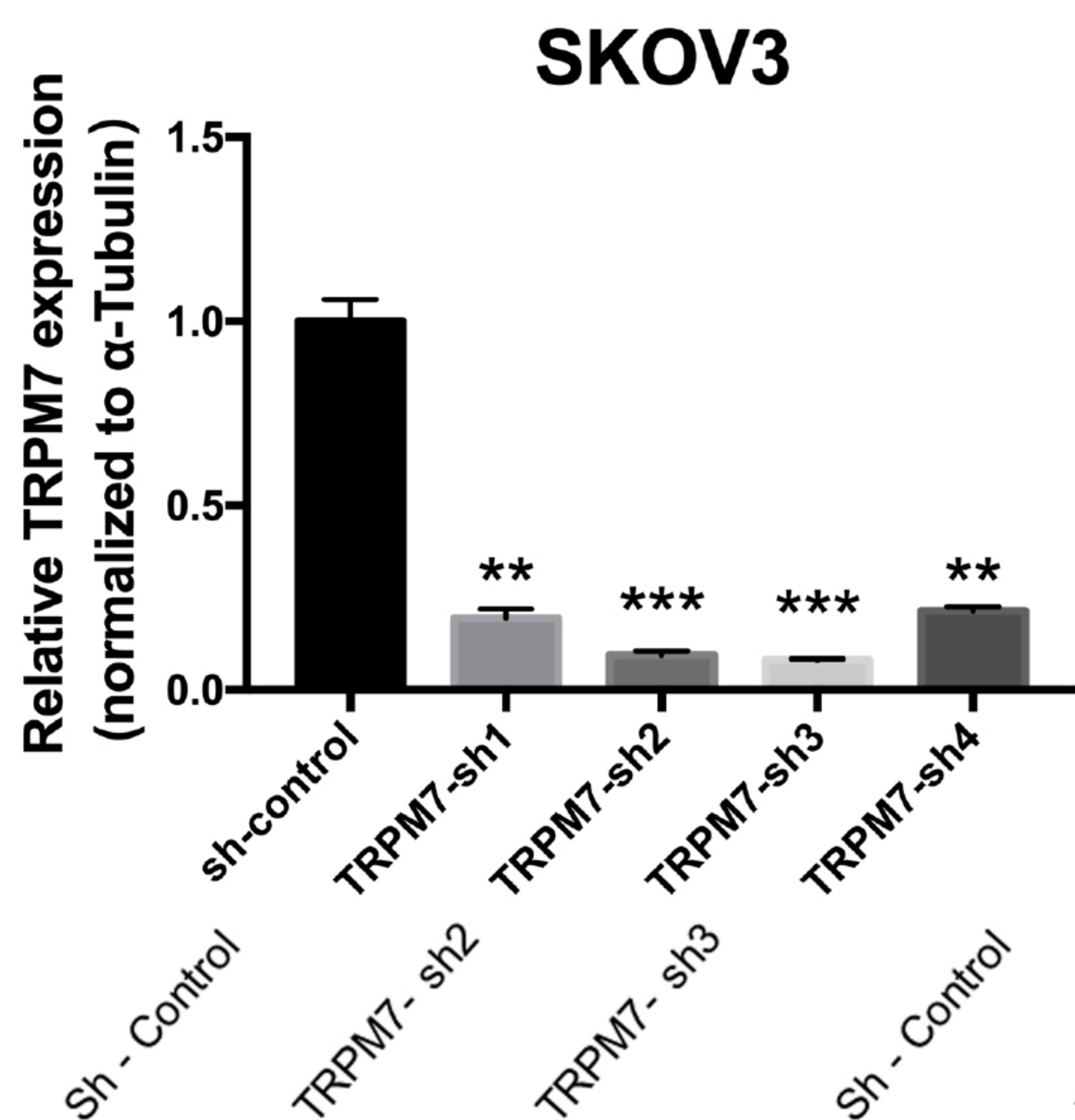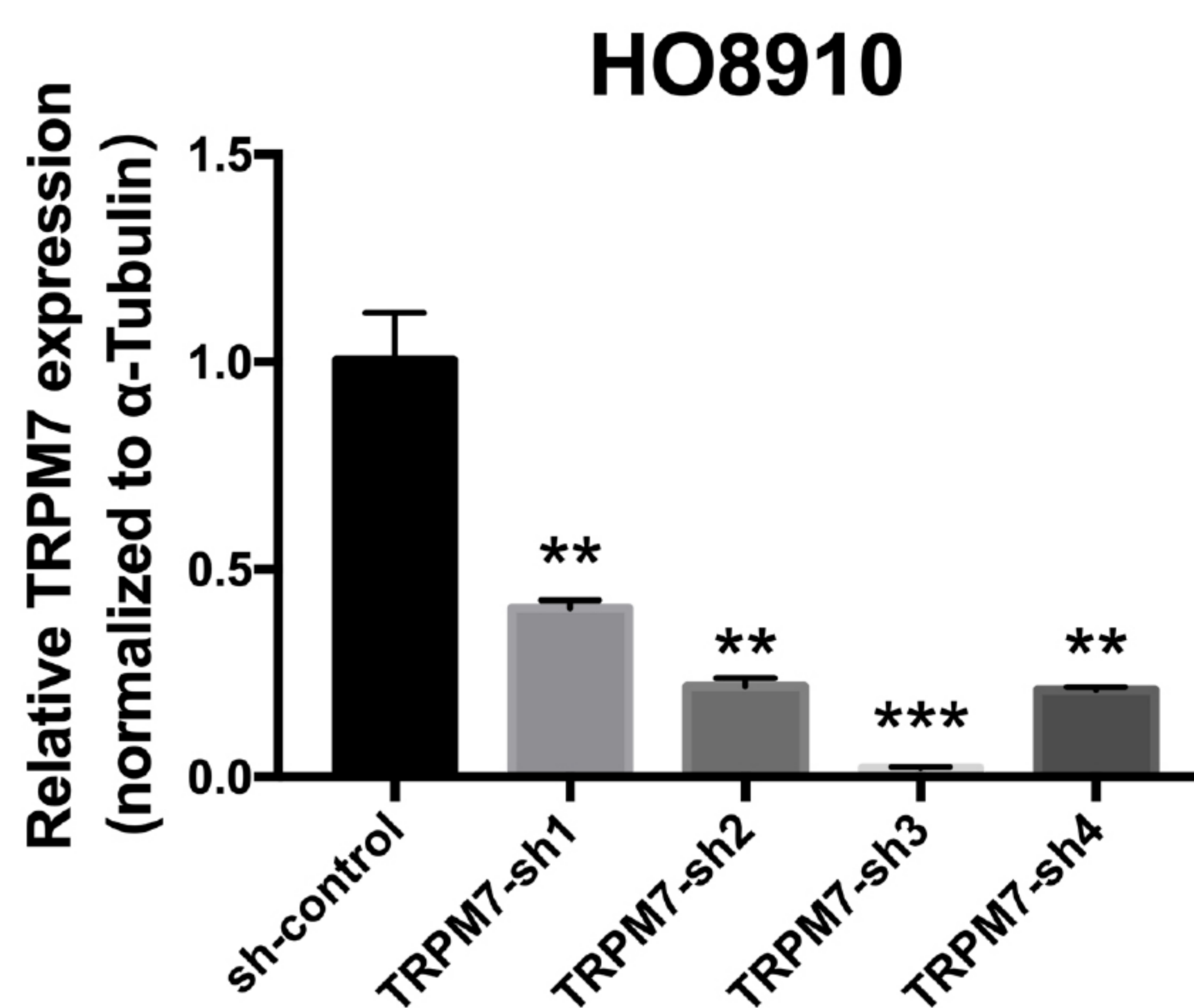**C**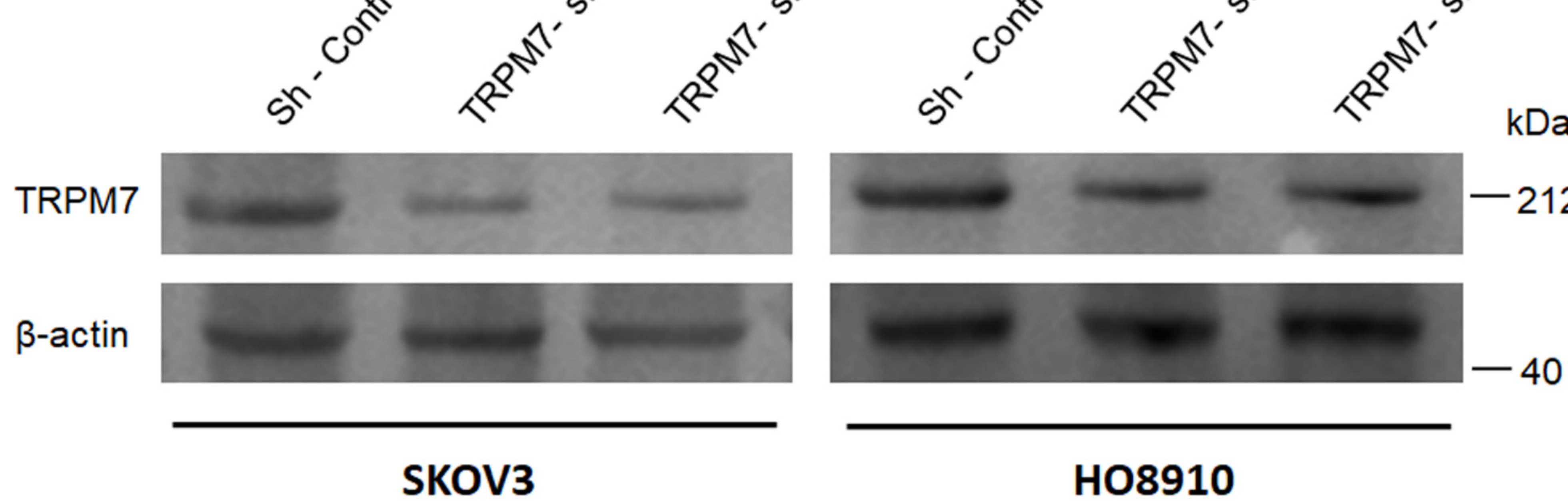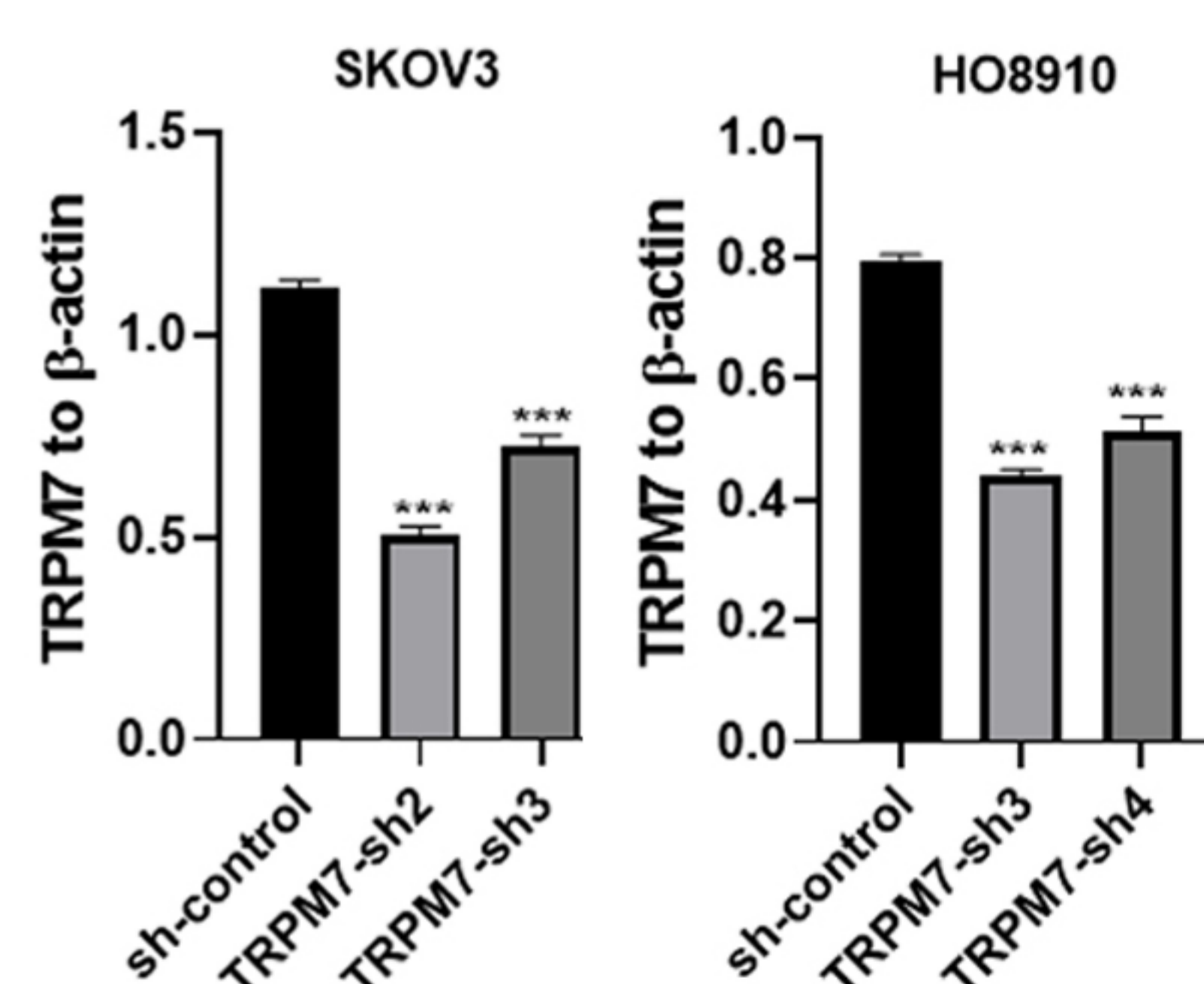**D**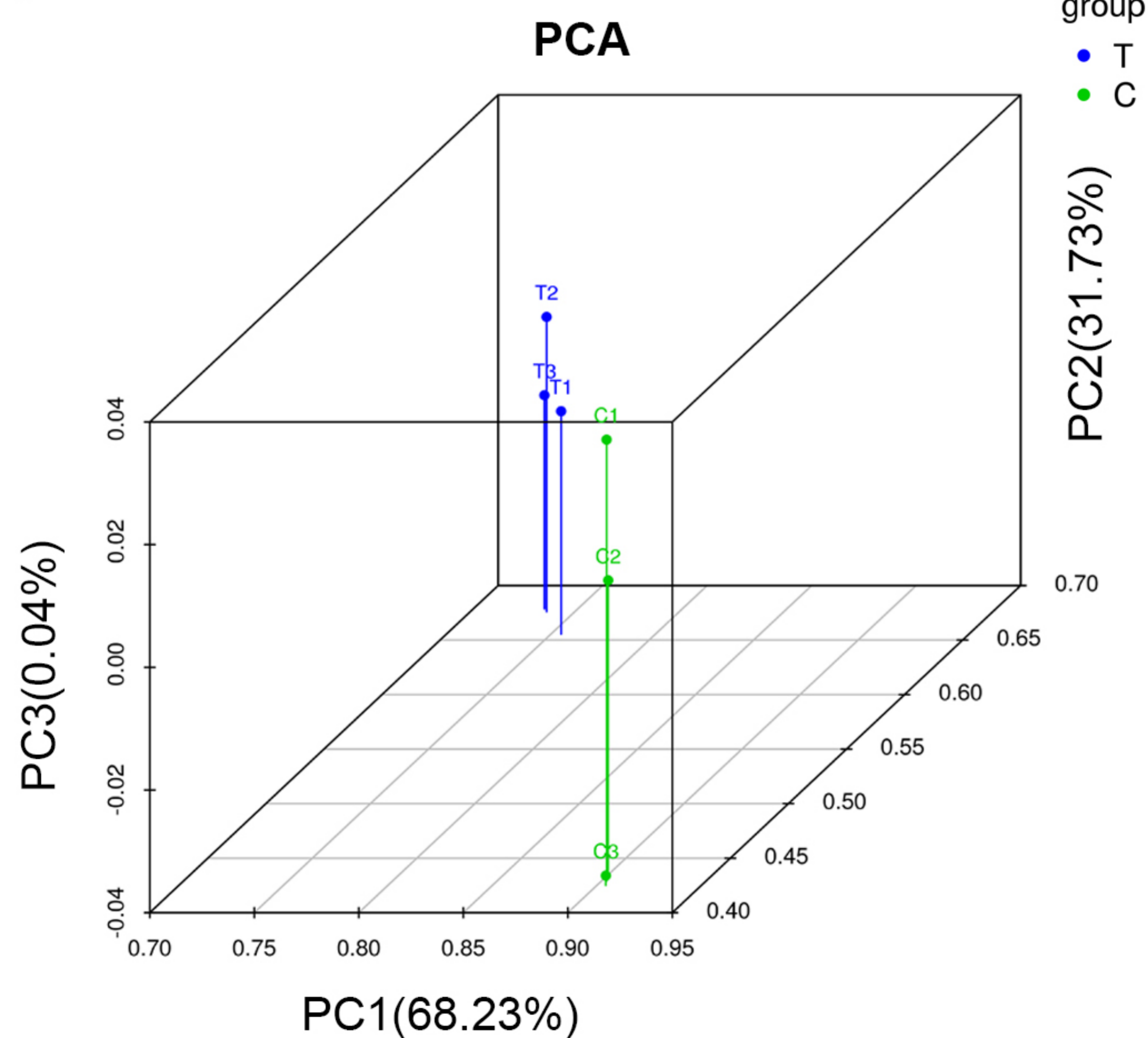**E**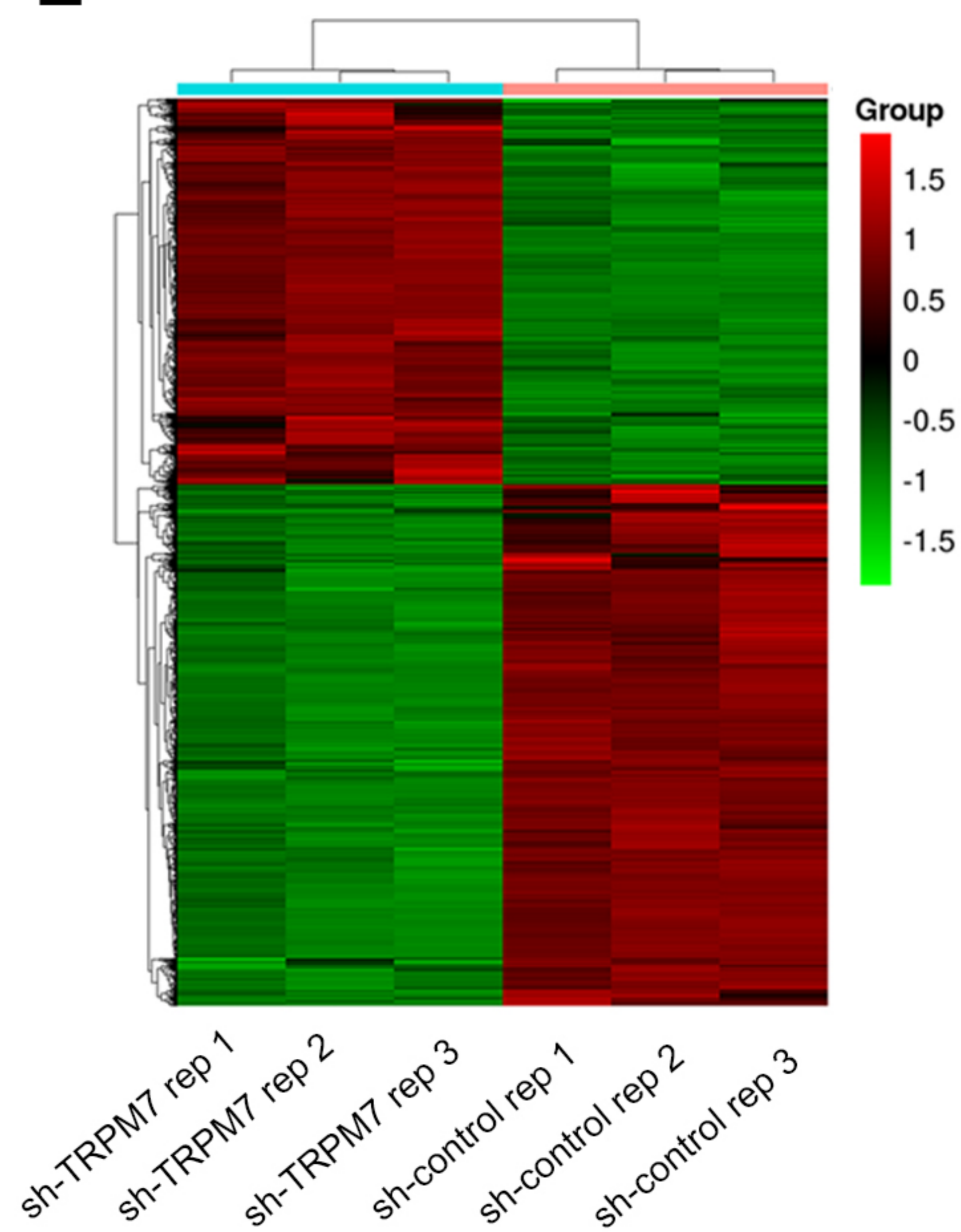

Supplement: Supplementary file 6 — Additional file 6: Supplementary Fig. 2. Verification of TRPM7 silencing in ovarian cancer cells. (A) Western blot analyses of TRPM7 expression in SKOV3 and HO8910 cells. SKOV3 and HO8910 cells were transduced with lentiviruses for expressing scramble RNA, TRPM7-sh1, TRPM7-sh2, TRPM7-sh3 or TRPM7-sh4, respectively. Four days later, the relative levels of TRPM7 expression in each group were quantified by RT-qPCR (B) and Western blot (C). (D) PCA score plot. (E) Heatmap displayed the DEGs between control and TRPM7 silencing SKOV3 cells. [file 13046_2022_2252_MOESM6_ESM.pdf]

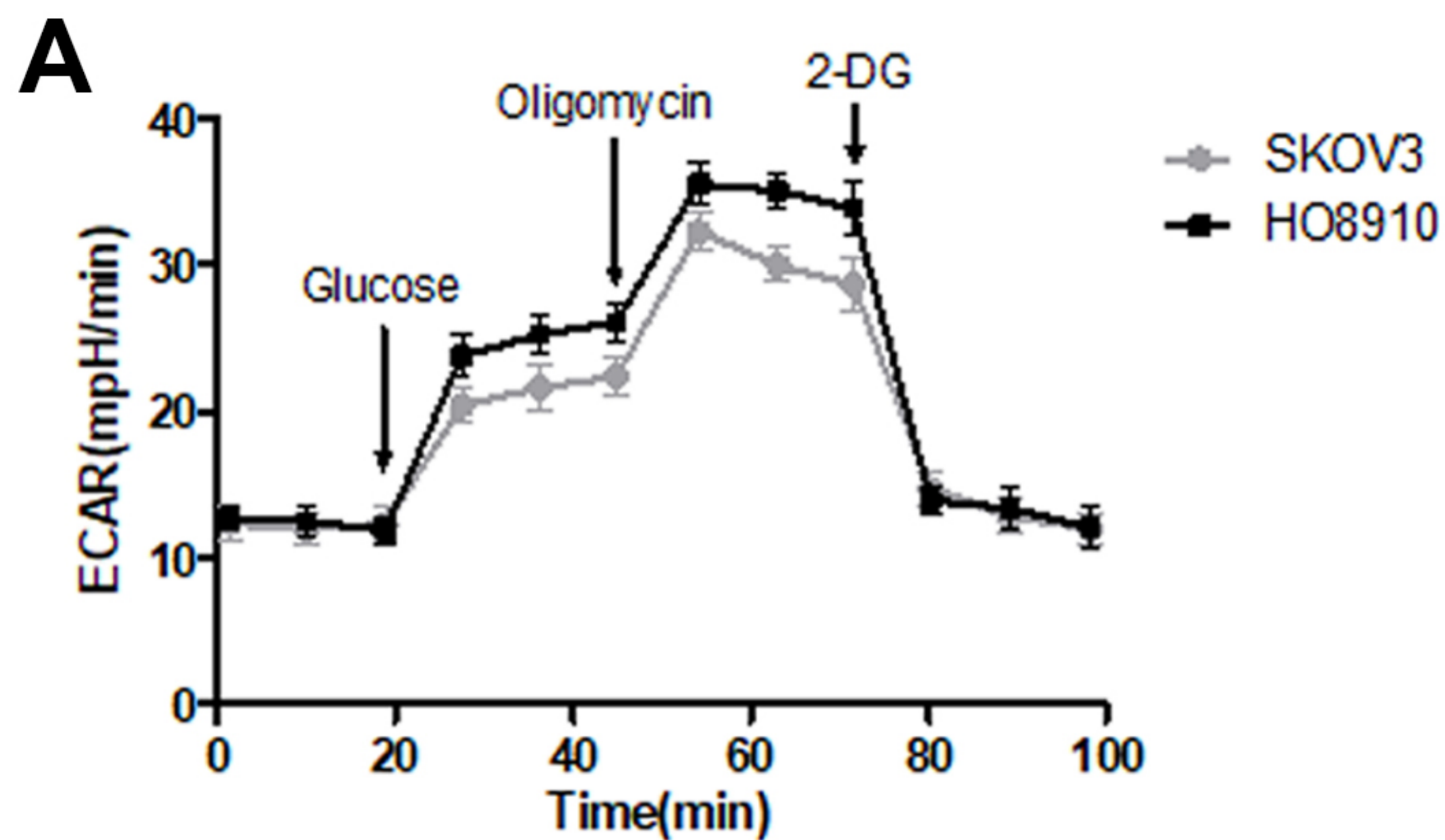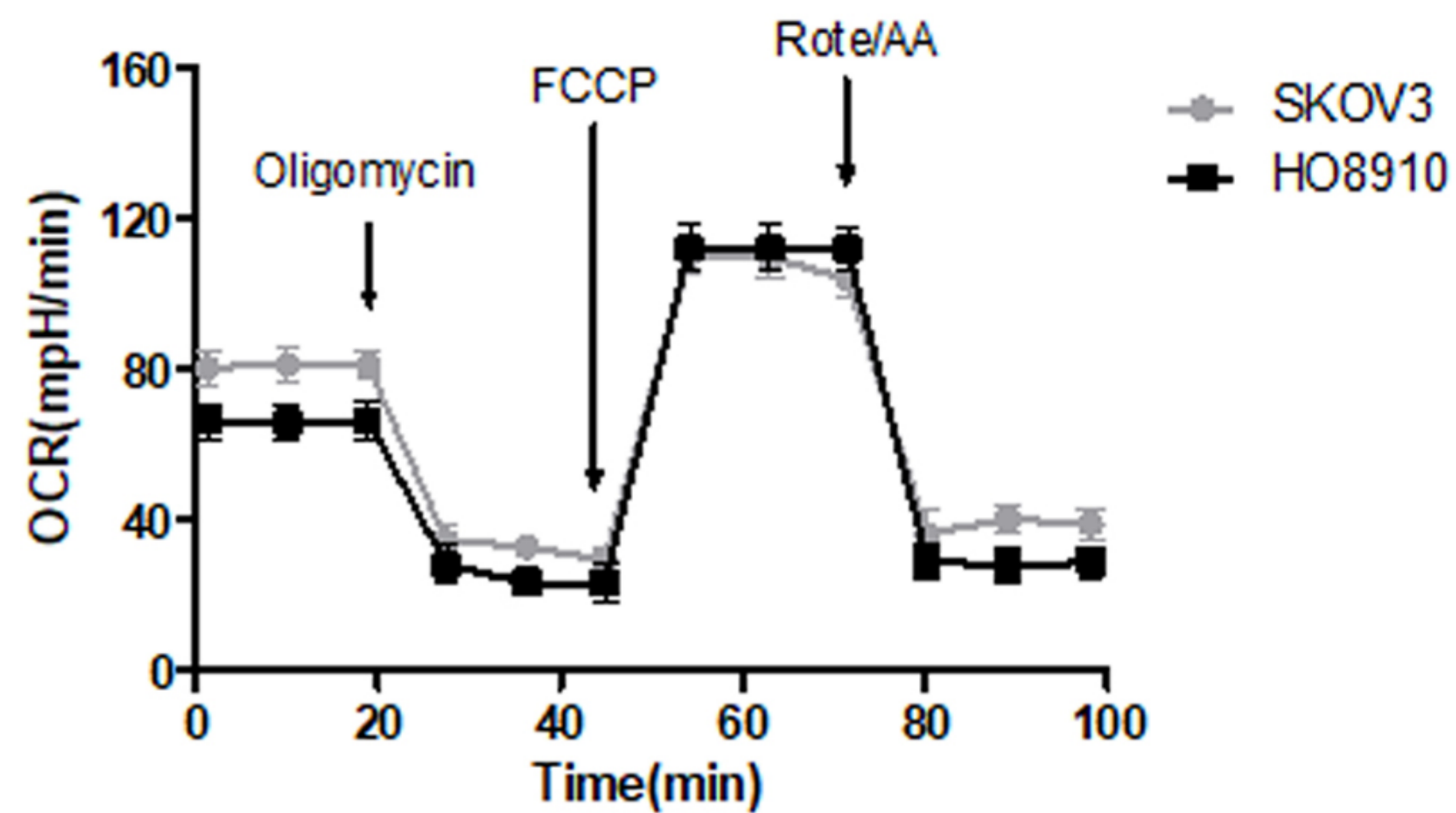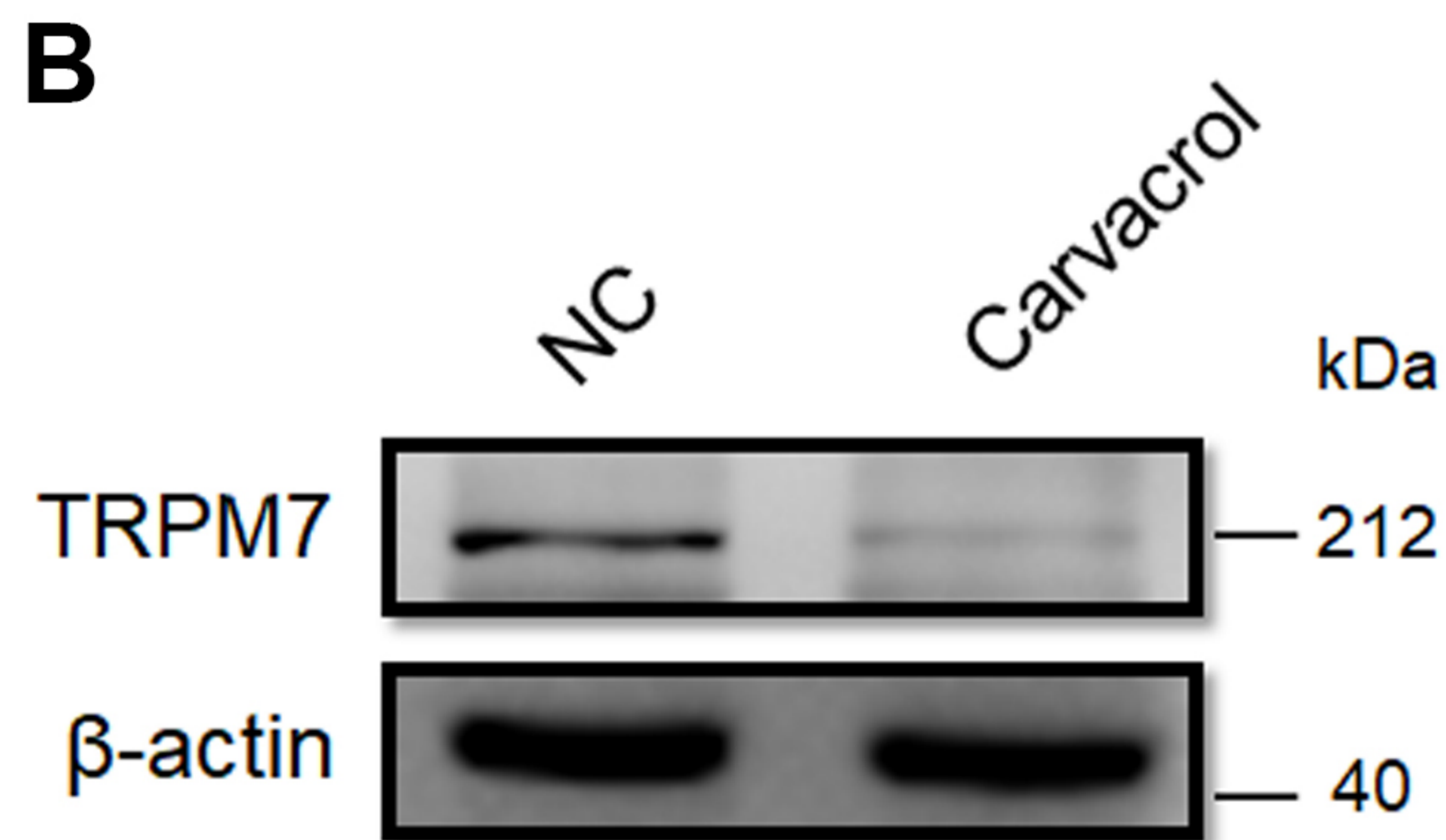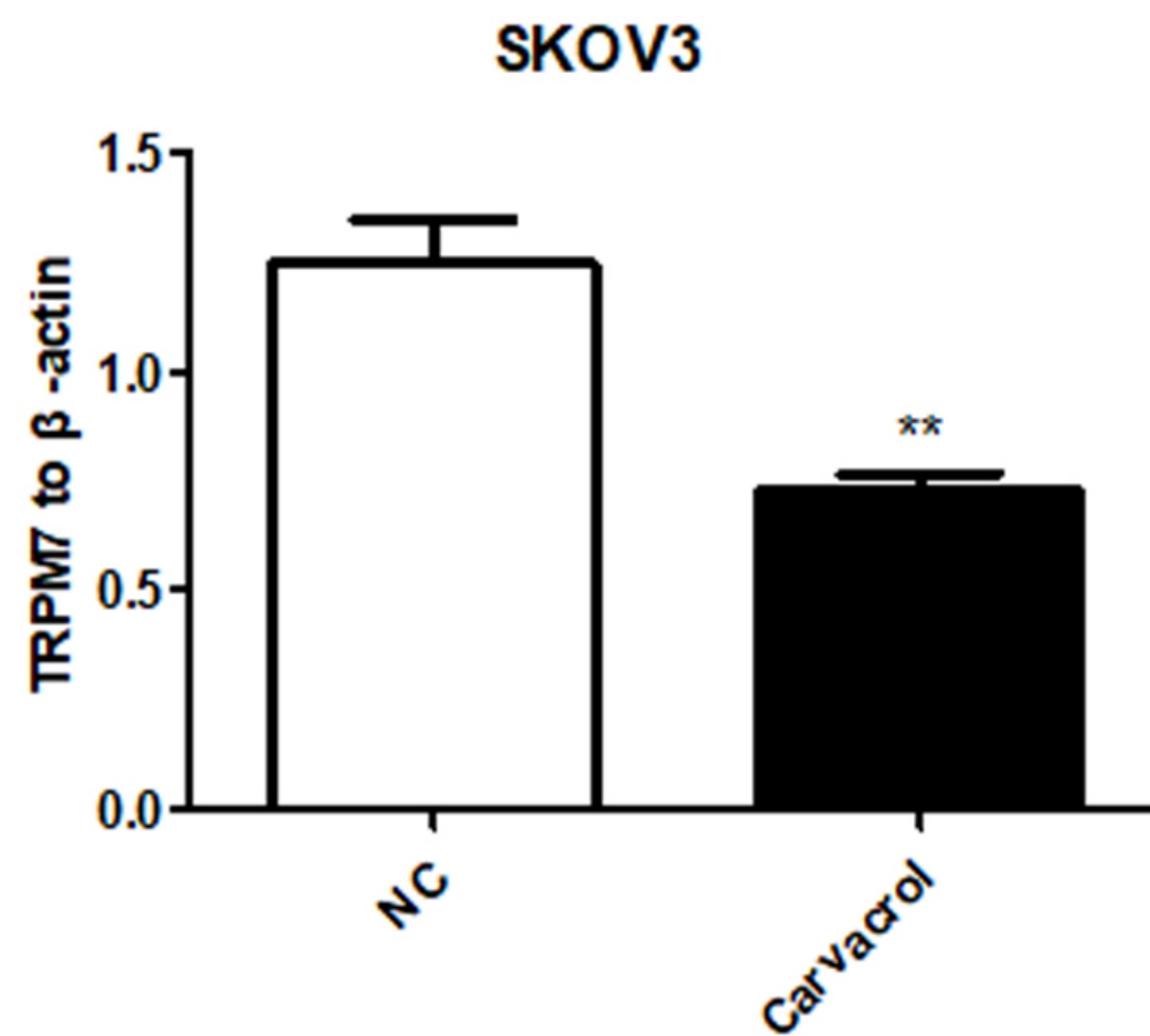

Supplement: Supplementary file 7 — Additional file 7: Supplementary Fig. 3. (A) ECARs and OCRs in SKOV3 and HO8910 cells. (B) Western blot analyses of TRPM7 expression in SKOV3 and HO8910 cells. (C) Western blot analysis of TRPM7 expression in SKOV3 cells following treatment with, or without, carvacrol. [file 13046_2022_2252_MOESM7_ESM.pdf]

A

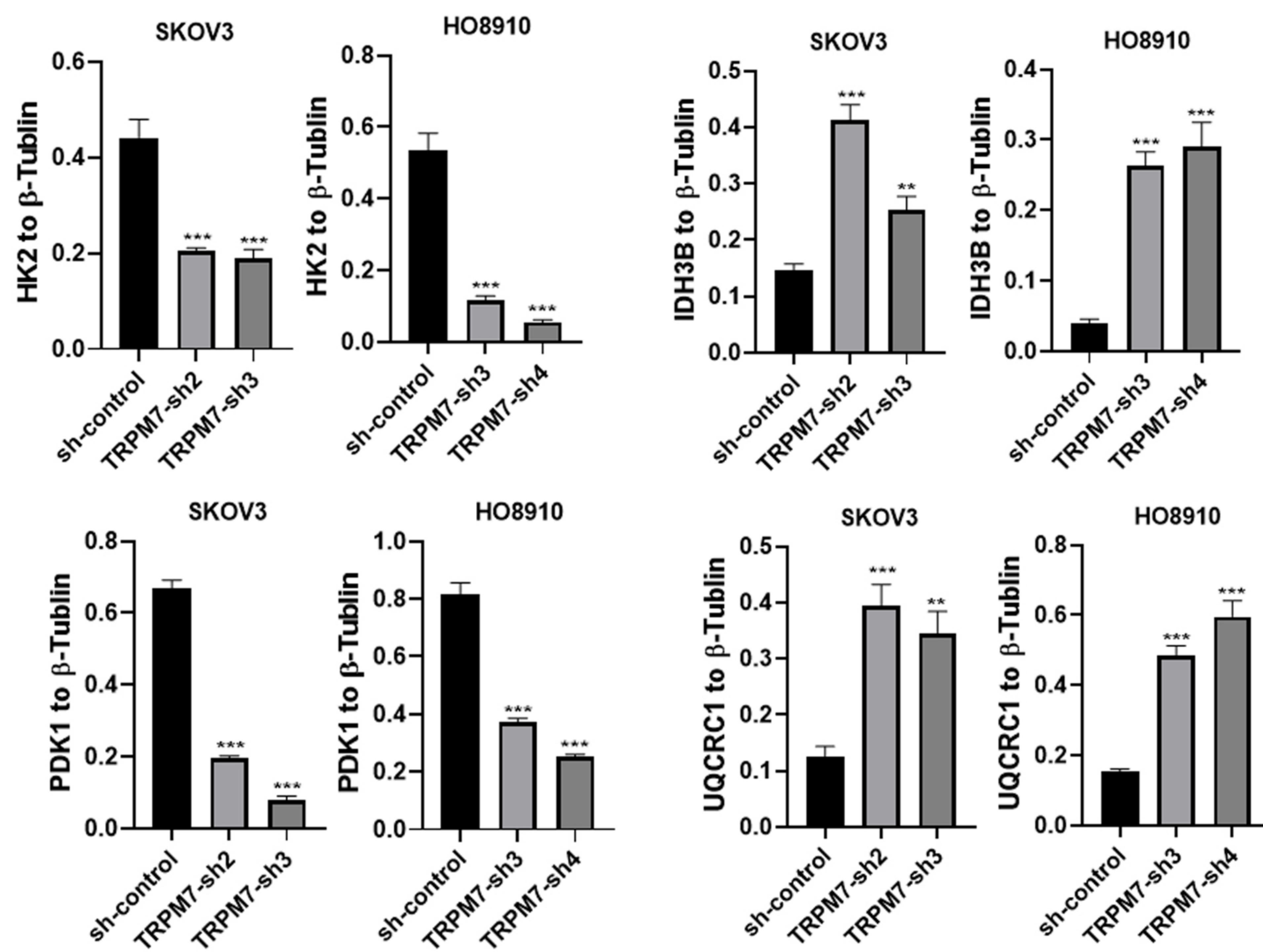

B

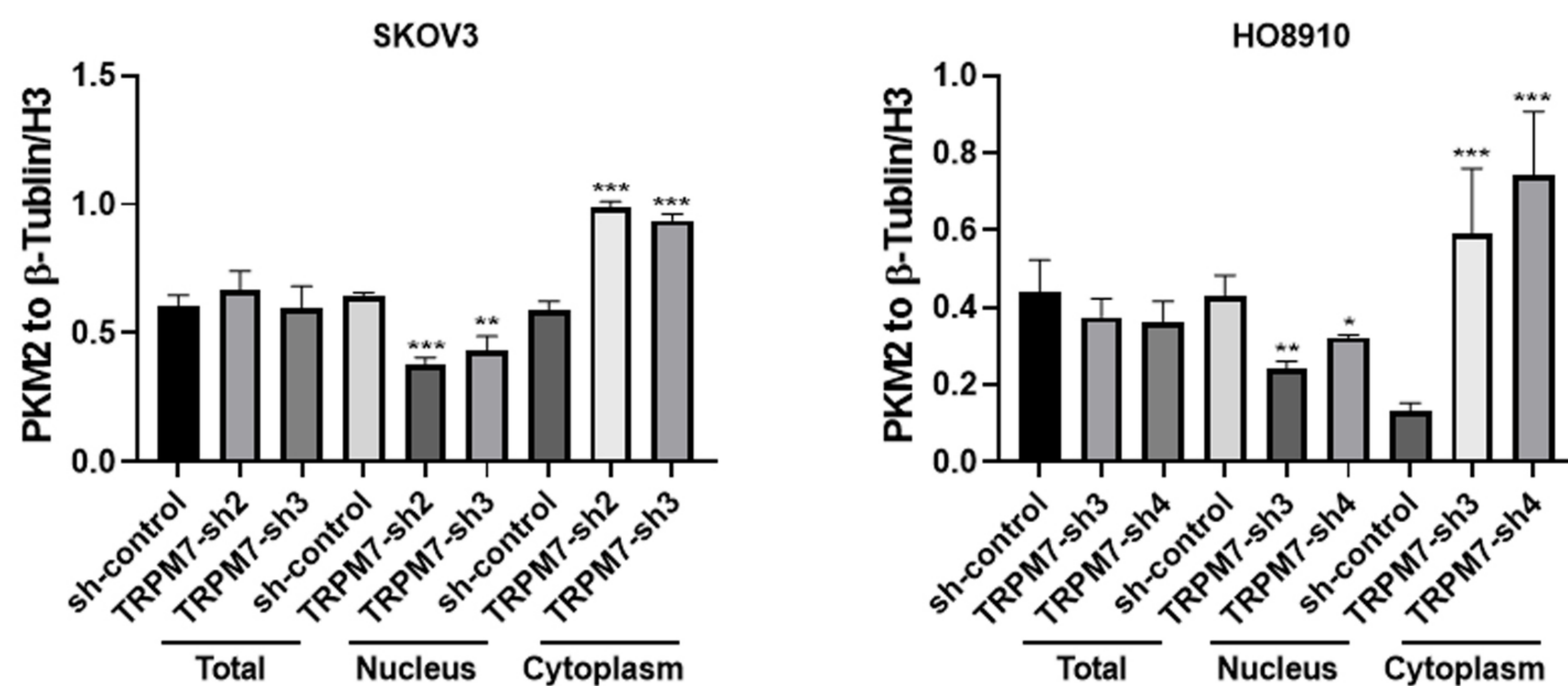

C

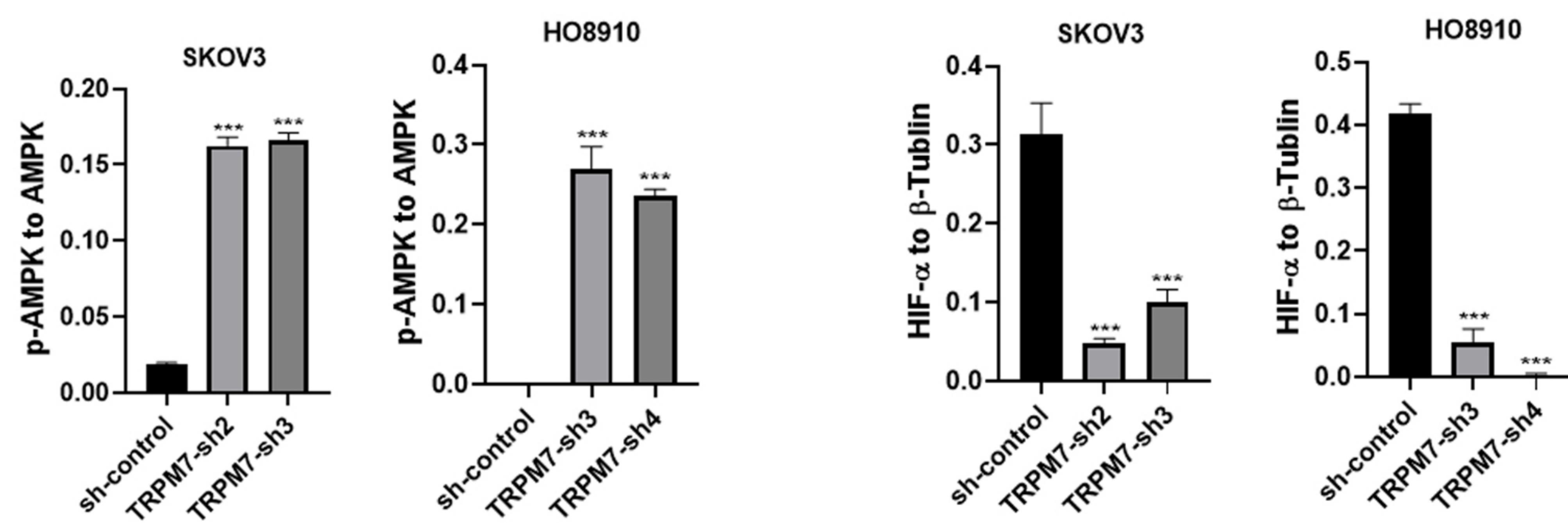

Supplement: Supplementary file 8 — Additional file 8: Supplementary Fig. 4. The quantitative analysis of Western blot data in Fig. 3. [file 13046_2022_2252_MOESM8_ESM.pdf]

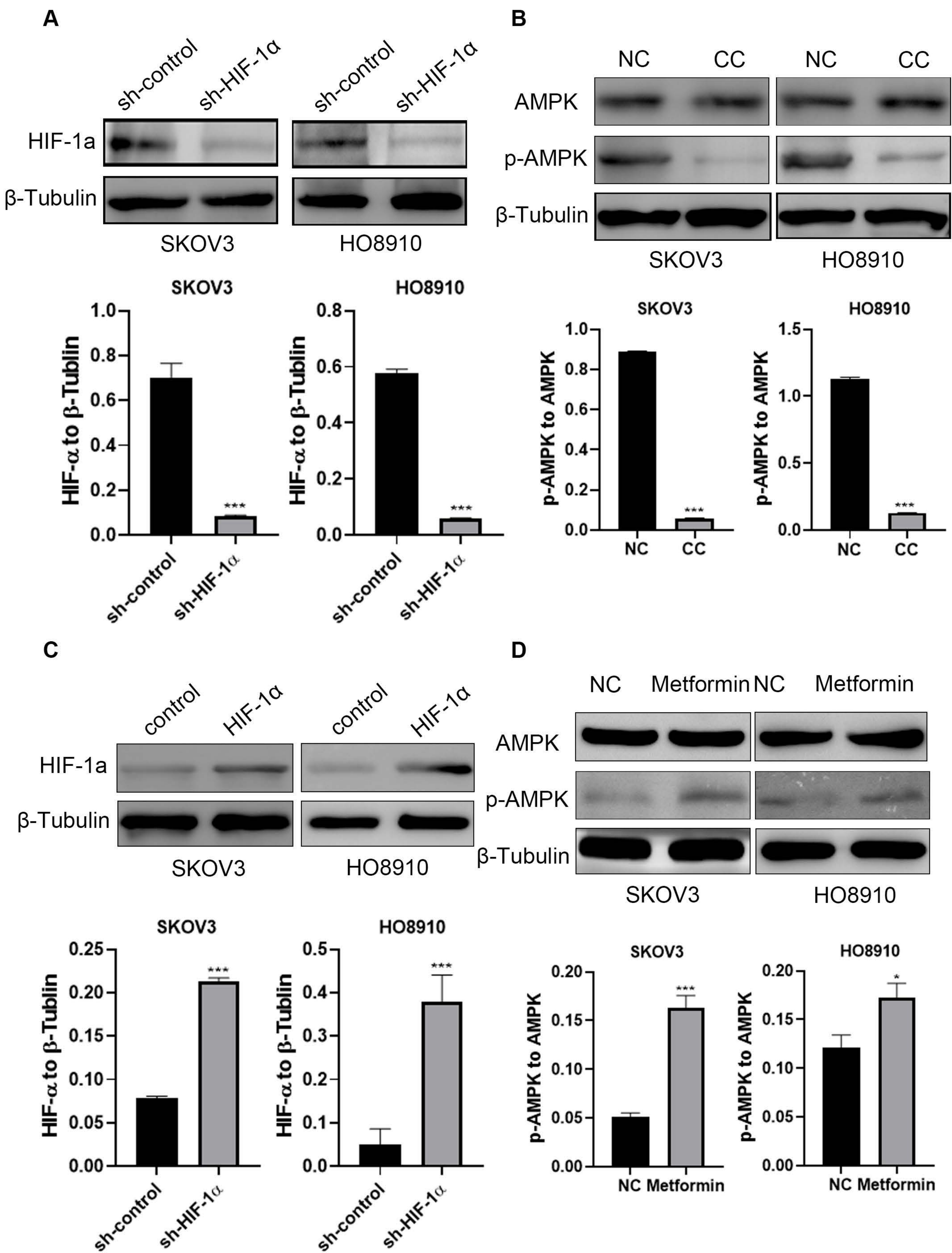

Supplement: Supplementary file 9 — Additional file 9: Supplementary Fig. 5. Verification of HIF-1α silencing and over-expression as well as modulating AMPK activation in ovarian cancer cells by Western blot. (A) Western blot analysis of HIF-1α silencing in SKOV3 and HO8910 cells. (B) Treatment with CC attenuated AMPK activation in SKOV3 and HO8910 cells. (C) Western blot analysis of HIF-1α over-expression in SKOV3 and HO8910 cells. (D) Treatment with metformin enhanced AMPK activation in SKOV3 and HO8910 cells. [file 13046_2022_2252_MOESM9_ESM.pdf]

A

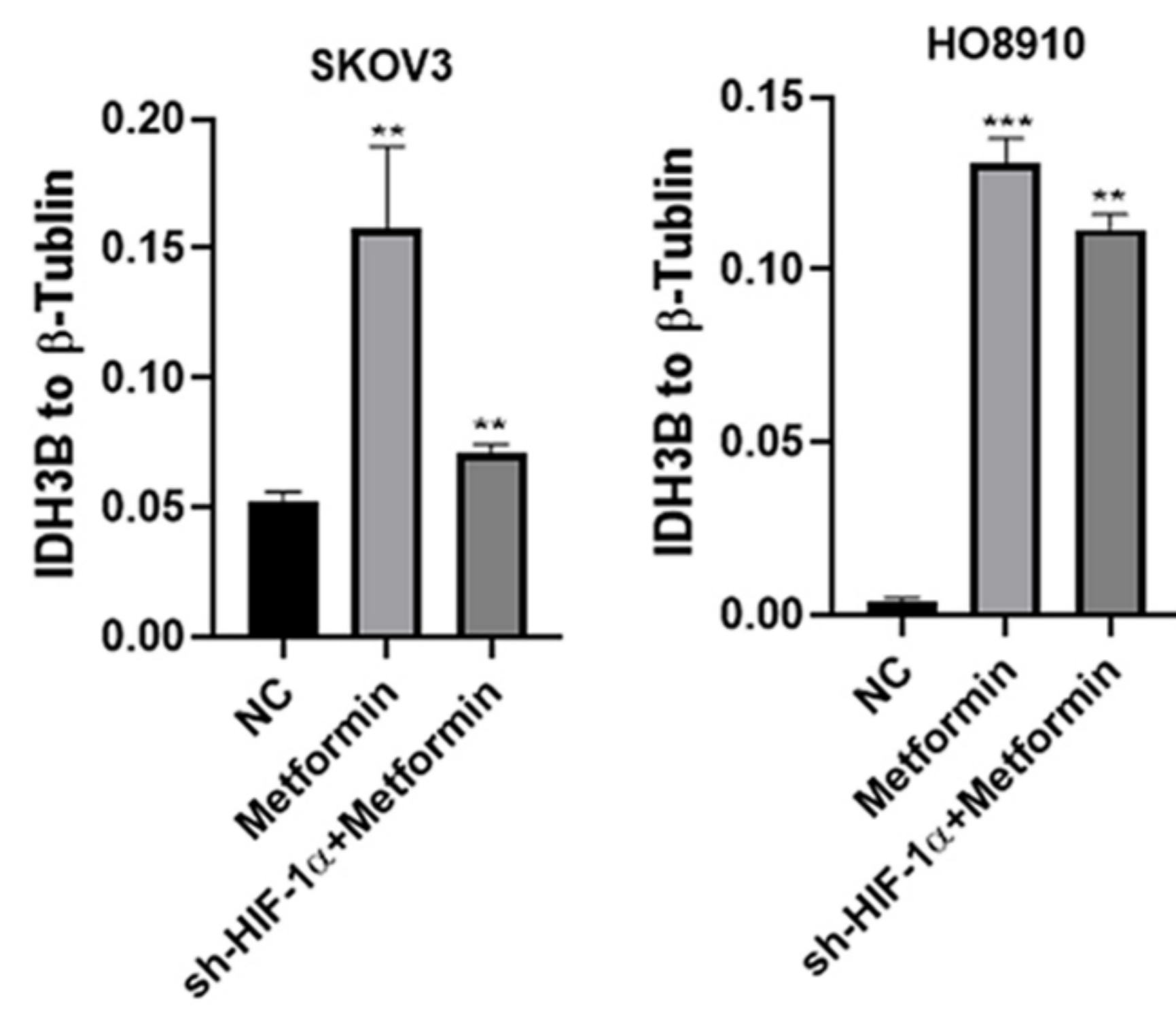

B

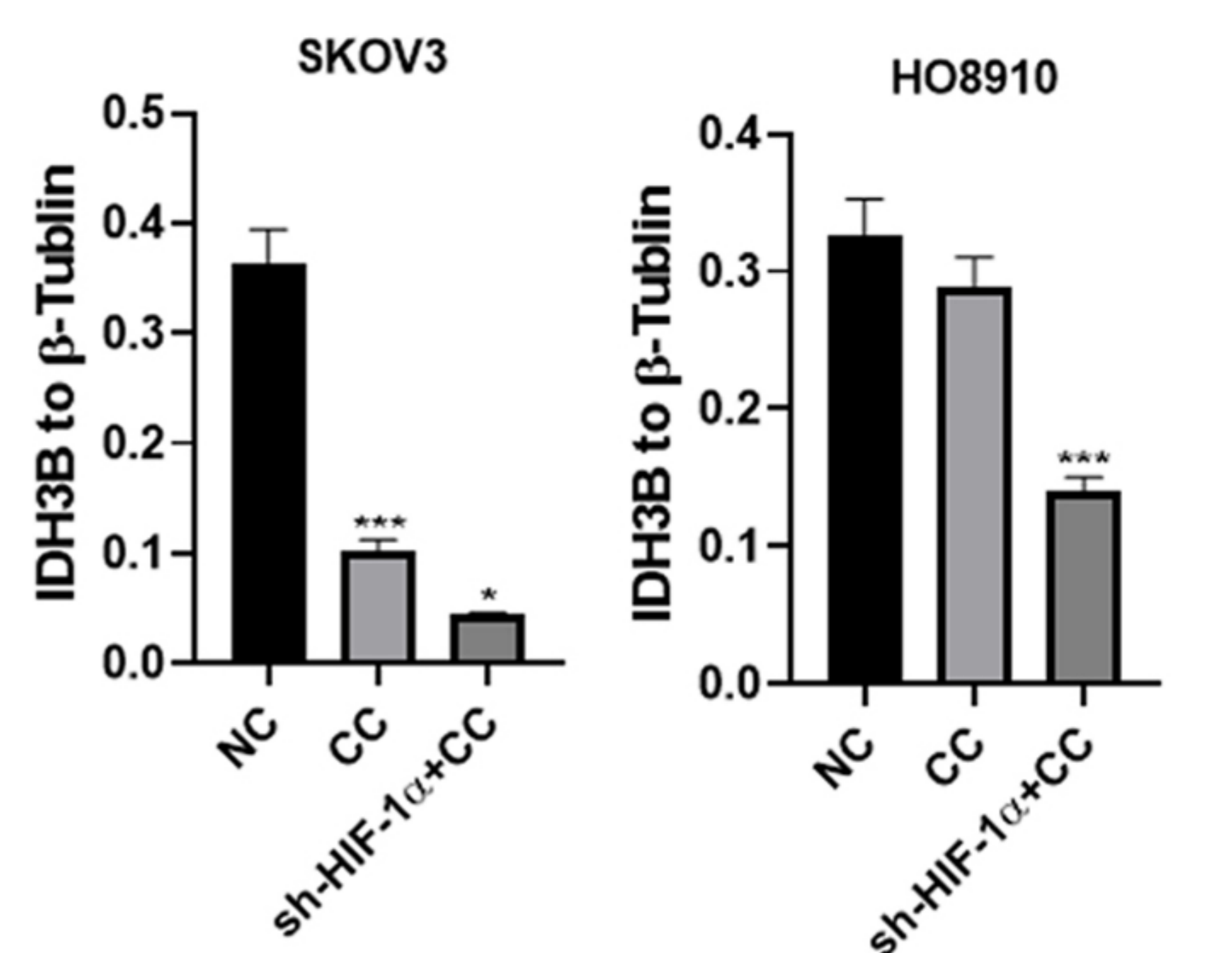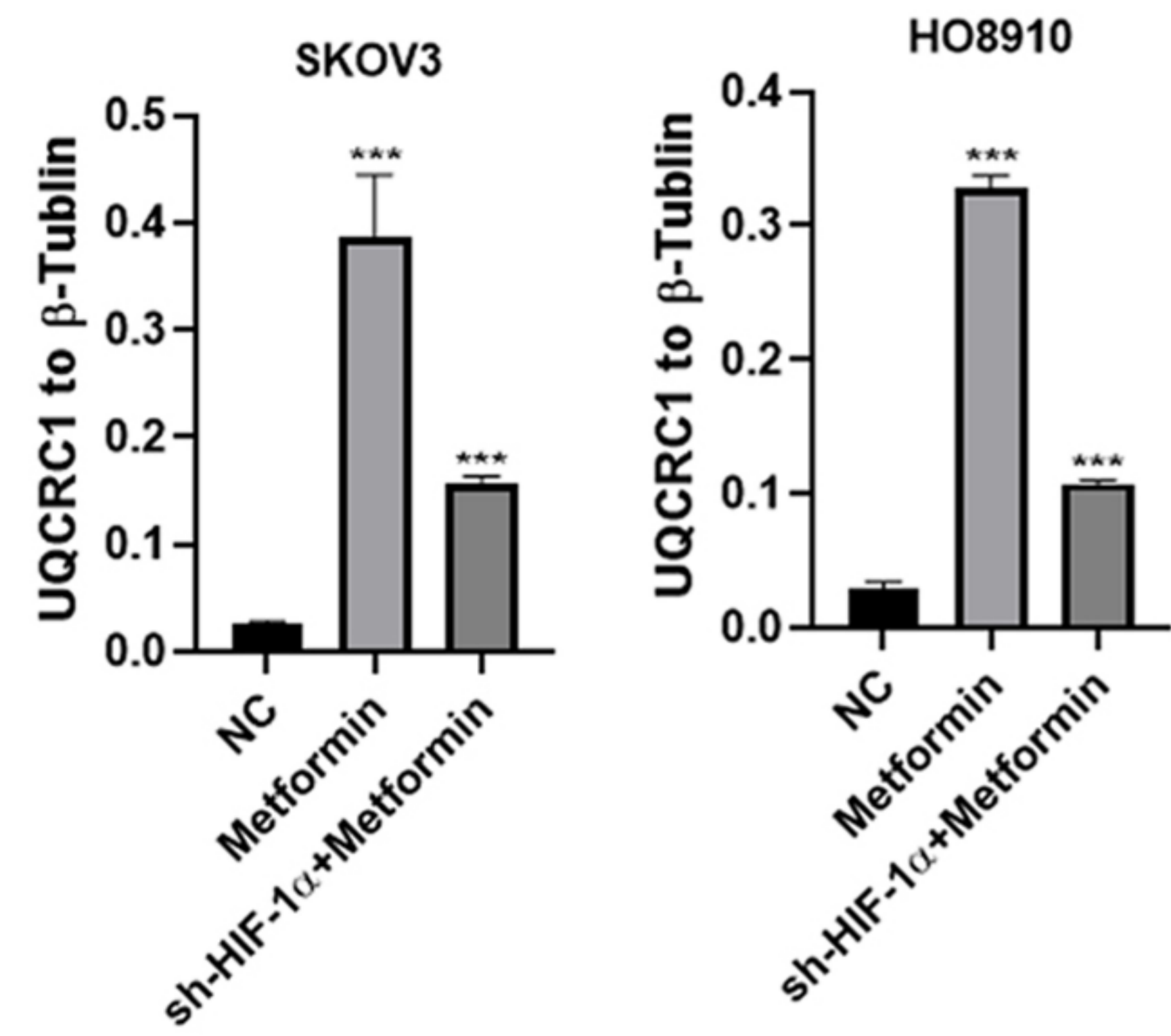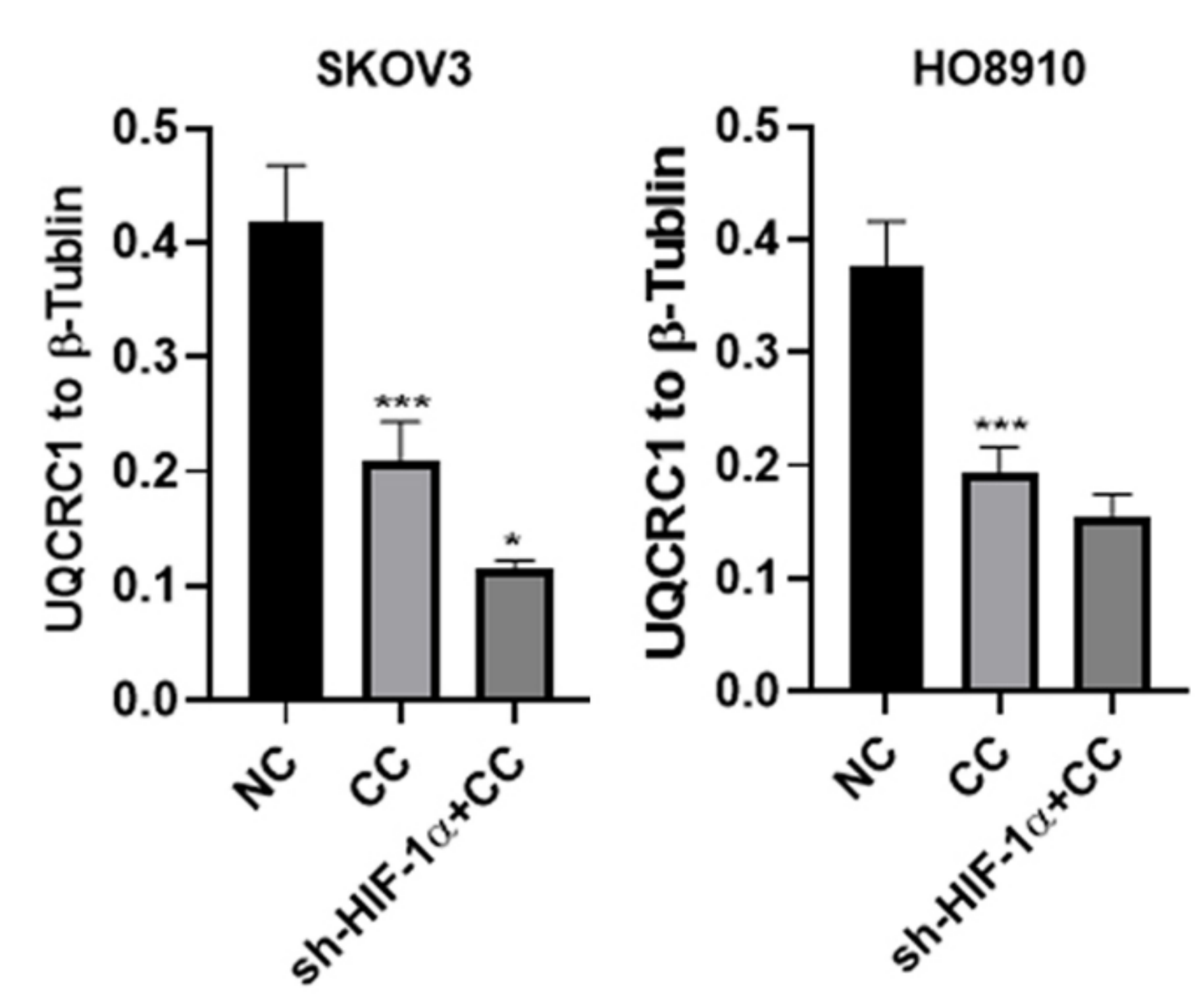

C

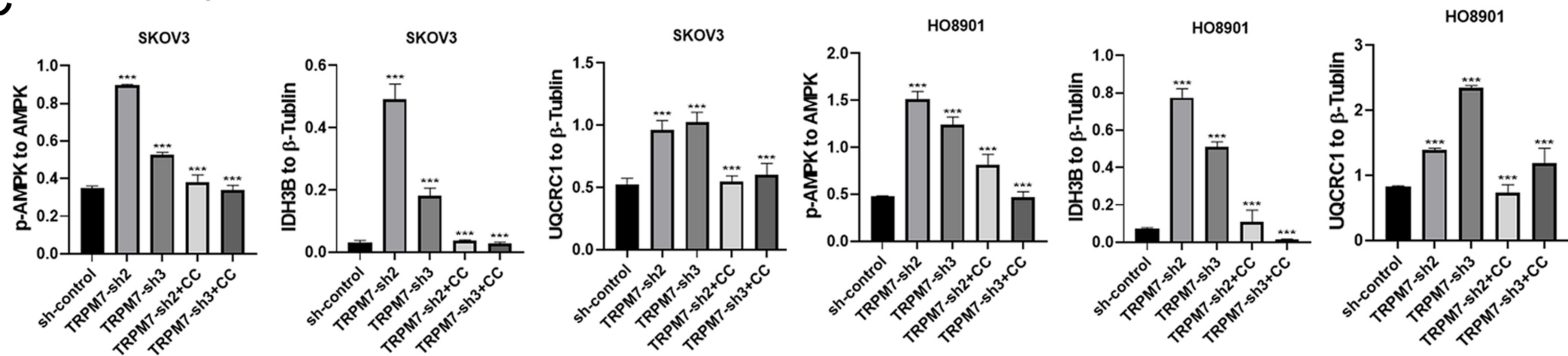

D

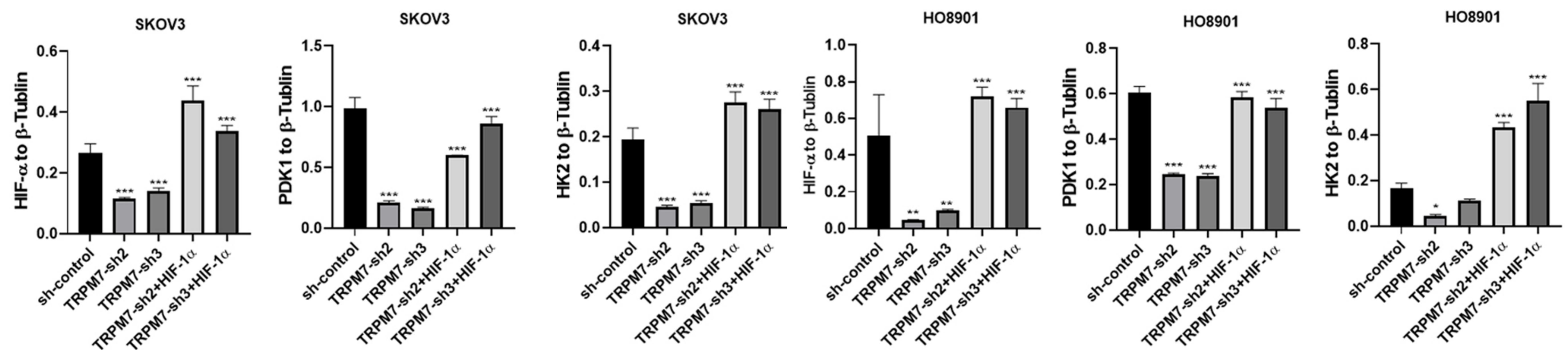

Supplement: Supplementary file 10 — Additional file 10: Supplementary Fig. 6. The quantitative analysis of Western blot data in Fig. 6. [file 13046_2022_2252_MOESM10_ESM.pdf]
